# LHPP-Mediated Histidine Dephosphorylation Suppresses the Self-Renewal of Mouse Embryonic Stem Cells

**DOI:** 10.3389/fcell.2021.638815

**Published:** 2021-03-16

**Authors:** Rong Mu Xia, Dong Bo Yao, Xue Min Cai, Xiu Qin Xu

**Affiliations:** Institute of Stem Cell and Regenerative Medicine, School of Medicine, Xiamen University, Xiamen, China

**Keywords:** embryonic stem cell, self-renewal, histidine dephosphorylation, LHPP, post-translational modification

## Abstract

Self-renewal of embryonic stem cells (ESCs) is orchestrated by a vast number of genes at the transcriptional and translational levels. However, the molecular mechanisms of post-translational regulatory factors in ESC self-renewal remain unclear. Histidine phosphorylation, also known as hidden phosphorylation, cannot be detected by conventional experimental methods. A recent study defined phospholysine phosphohistidine inorganic pyrophosphate phosphatase (LHPP) as a histidine phosphatase, which regulates various biological behaviors in cells via histidine dephosphorylation. In this study, the doxycycline (DOX)-induced hLHPP-overexpressing mouse ESCs and mouse LHPP silenced mESCs were constructed. Quantitative polymerase chain reaction (qPCR), western blotting analysis, immunofluorescence, Flow cytometry, colony formation assays, alkaline phosphatase (AP) and bromodeoxyuridine (Brdu) staining were performed. We found that the histidine phosphorylation level was strikingly reduced following LHPP overexpression. Besides, the expression of *Oct4* and *Lefty1*, indispensable genes in the process of ESCs self-renewal, was significantly down-regulated, while markers related to the differentiation were markedly elevated. Moreover, LHPP-mediated histidine dephosphorylation induced G_0/_G_1_ phase arrest in mESCs, suggesting LHPP was implicated in cell proliferation and cell cycle. Conversely, silencing of *Lhpp* promoted the self-renewal of mESCs and reversed the RA induced increased expression of genes associated with differentiation. Mechanistically, our findings suggested that the enzymatic active site of LHPP was the cysteine residue at position 226, not 53. LHPP-mediated histidine dephosphorylation lowered the expression levels of β*-catenin* and the cell cycle-related genes *CDK4* and *CyclinD1*, while it up-regulated the cell cycle suppressor genes *P21* and *P27*. Taken together, our findings reveal that LHPP-mediated histidine dephosphorylation plays a role in the self-renewal of ESCs. LHPP-mediated histidine dephosphorylation inhibited the self-renewal of ESCs by negatively regulating the Wnt/β-catenin pathway and downstream cell cycle-related genes, providing a new perspective and regulatory target for ESCs self-renewal.

## Introduction

Embryonic stem cells (ESCs), with the attributes of pluripotency and self-renewal, have the ability to differentiate into multicell lineages (Dogan, 2018). Mounting evidence has shown that ESCs self-renewal is orchestrated by a variety of transcription factors, signaling molecules and RNA regulatory factors (Huang et al., 2015; Abu-Dawud et al., 2018). Previous work has indicated that transcriptional and post-transcriptional modifications are important for the regulation of ESCs self-renewal (Li et al., 2014). It has been reported that Oct4 and NANOG recruit LSD1 and bind to promoters of development-related genes, thereby controlling ESCs self-renewal by modulating H3K4 and H3K27 methylation (Adamo et al., 2011; Whyte et al., 2012). Zinc finger protein ZC3H13 maintains ESCs self-renewal via m6A modifications of mRNA (Wen et al., 2018). In addition, Foxp1-mediated ESCs-specific splicing switches play a crucial role in maintaining self-renewal (Gabut et al., 2011).

Translational regulation and post-translational modifications have a significant impact on ESCs self-renewal (Cassar and Stanford, 2012), where parsimonious translation in the pluripotent state and hierarchical translational regulation during differentiation control self-renewal and determine the fate of ESCs (Sampath et al., 2008). The ubiquitin proteasome pathway is responsible for most of the protein degradation that regulates ESCs self-renewal (Liu et al., 2016; Zhao et al., 2017). Additionally, phosphorylation is the basis for the activation and functional accuracy of a great number of proteins, and is also an important mechanism for the regulation of post-translational temporal and spatial distribution (Watanabe and Osada, 2016; Taracha et al., 2017). Previous studies have primarily focused on the phosphorylation of serine, threonine and tyrosine residues. Protein phosphorylation is related to ESCs self-renewal (Wang et al., 2016). LIF-induced activation of the JAK/STAT3, PI3K/AKT, and SHP2/MAPK pathways facilitates the nuclear translocation of signal factors, leading to gene expression that promotes ESCs self-renewal. Since its discovery, protein phosphorylation has been revealed to play a vital role in drug development, disease pathogenesis and treatment. However, the molecular mechanisms of the maintenance of ESCs self-renewal at the post-translational level remain unclear.

Histidine residues can also be phosphorylated (Boyer et al., 1962). However, histidine phosphorylation, also known as “hidden phosphorylation,” is characterized by an unstable P-N bond, and increased sensitivity to heat and acids. Phosphorylated histidine exists as two isomers, 1-phosphate histidine (1-pHis) and 3-phosphate histidine (3-pHis), which are produced by the phosphorylation of N1 and N3 on histidine residues, respectively (Fuhs et al., 2015). Previous studies have found that LHPP has been defined as a histidine phosphatase that regulates biological behavior in cells via histidine dephosphorylation (Hindupur et al., 2018). In our previous work (unpublished data), during the myocardial differentiation of ESCs and RA-induced differentiation, the expression of *Lhpp* was significantly upregulated. Meanwhile, compared with ESCs, the expression levels of *Lhpp* in various adult mouse tissues were upregulated to a much greater degree. However, whether LHPP-mediated histidine dephosphorylation affects ESCs self-renewal remains largely unknown. We hypothesized that LHPP-mediated histidine dephosphorylation may regulate the self-renewal of stem cells. Via construction of an inducible mouse ESCs (mESCs) overexpressing LHPP, and a lentivirus-mediated *Lhpp* silenced mESCs, we found that LHPP-mediated histidine dephosphorylation suppresses the self-renewal of mESCs, possibly by modulating the Wnt/β-catenin pathway, which provides a new perspective and regulatory target for understanding ESCs self-renewal.

## Materials and Methods

### Cell Culture

A2Lox-Cre mouse embryonic stem cells (mESCs) which is kindly donated by Kyba et al. (2002; Iacovino et al., 2014) were seeded onto an irradiated MEFs at the density of 5 × 10^5^ cells. R1 mESCs were obtained from the American Type Culture Collection (ATCC). A2Lox-Cre mESCs and R1 mESCs were cultured in DMEM (Gibco, Grand Island, NY, United States) containing 15% FBS (Hyclone, Utah, United States), 0.1 mM non-essential amino acids (Gibco, Grand Island, NY, United States), 1 mM sodium pyruvate (Gibco, Grand Island, NY, United States), 0.1 mM β-mercaptoethanol (Sigma-Aldrich, Saint Louis, MO, United States), 100 U/ml penicillin (Gibco, Grand Island, NY, United States), 100 μg/ml streptomycin (Gibco, Grand Island, NY, United States) and 1000 U/ml leukemia inhibitory factor (LIF) (ESGRO, Millipore, Chemicon, United States). The cells were incubated at 37°C (5% CO_2_) in a humidified incubator, and the culture medium was changed every 24 h.

### Construction of a Doxycycline (DOX)-Induced Human LHPP-Overexpressing Mouse ESC Line

In this study, the construction of a Doxycycline (DOX)-induced human LHPP-overexpressing mouse ESC line was performed as described previously (Zhang et al., 2016). Briefly, human *Lhpp* (h*Lhpp*) was sequenced and cloned into the p2Lox-Rbm24-Flag-IRES-GFP plasmid to replace Rbm24, which resulted in the p2Lox-hLHPP-IRES-GFP recombinant plasmid. To induce the expression of Cre recombinant enzyme prior to electroporation, the cells were cultured in mouse ESC medium containing 1 μg/ml DOX. The next day, the cells were adjusted to a concentration of 1 × 10^6^ cells per sample and resuspended in 90 μl Opti-MEM (Gibco, Grand Island, NY, United States). The Opti-MEM was equilibrated at room temperature before use. 10 μl p2Lox-hLHPP-IRES-GFP plasmid (10 μg) was used for each sample, which was transferred into a sterile cuvette and electroporated using the NEPA21 Super Electroporator (NEPA GENE, Japan). The procedure was conducted using the following parameters: 130 V, 5 ms; three pulses. Afterward, the cuvettes were immediately placed on ice for 5 min. The mixture was then transferred to a plate containing MEFs. Two days after inoculation, the cells were screened with 300 μg/ml of G418 for a further 7 days, and single clones were selected for amplification and identification.

### Construction of a Mouse *Lhpp* Silenced mESC Line

Short hairpin RNA (shRNA) targeting Lhpp was used to construct LHPP-silenced ESC line. Briefly, sh1 Lhpp sequence (5′-TGGGAAAAGGACGCTATTACAAG-3′); sh2 Lhpp sequence (5′-GCTCAGAATTTGATCAGAT-3′); sh3 Lhpp sequence (5′-GGGAAAAGGACGCTATTACAAGG-3′) was cloned into lentivirus plasmid (plvx), and then transfected into A2Lox-Cre mESCs, followed by incubation with 10 μg/mL puromycin for 3 days. Finally, shLHPP-A2Lox mESCs was validated by qPCR and western blotting. Besides, R1 mESCs infected the above virus were subjected to the same treatment to obtain LHPP-silenced R1 mESCs.

### Construction of a Human LHPP-Overexpressing R1 mESCs

To construct R1 mESCs overexpressing human LHPP, the CDS region of human *Lhpp* was cloned into the polyclonal site of the plvcs plasmid as described above and transfected into 293T cells together with a lentiviral packaging plasmid to produce lentiviral particles. Afterward, R1 mESCs were infected using these lentiviruses. Forty-eight hours later, the cells were selected with 10 μg/mL of puromycin for 3 days to generate stable cell lines. The lentivirus vector carrying a non-targeting shRNA sequence was used as a control.

### Construction of LHPP Overexpressing Plasmid and the Mutant Plasmids

To construct the plasmid for transient overexpression of *Lhpp*, the CDS region of *Lhpp* was cloned into the pXJ-40 plasmid with the following primers. *Lhpp* upstream primer (*Xho*I restriction enzyme site): 5′-CCGCTCGAGATGGCACCGTGGGGCAAG-3′; *Lhpp* downstream primer (Pst1 restriction enzyme site): 5′-GCTGCAGCTTGTCGGCGTGCTGCAGC-3′. Subsequently, to construct plasmid contained a point mutation in the *Lhpp* sequence, the mutant *Lhpp* (C53S) and *Lhpp* (C226S) were generated using forward primer and reverse primer carrying the mutation, respectively. The primers were as follows: h*Lhpp* (c.158G > C) forward: TGAAGGTGAGGTTCTcCACCAACGAG, h*Lhpp* (c.158G > C) reverse: gAGAACCTCACCTTCAGCCGGGAAC; h*Lhpp* (c.677G > C) forward: GCGGTGCCCAGCGGTcTGGAAT GAGA, h*Lhpp* (c.677G > C) reverse: gACCGCTGGGCACCG CCGACGTCGCC. The products were digested with DMT enzyme for 1 h at 37°C, and then transformed into *Escherichia coli* for amplification. Mutagenesis was detected by Sanger sequencing. After successful construction of the plasmids, the plasmid vectors were transfected into 293T cells. Forty-eight hours later, western blotting assay was carried out to detect the intracellular pHis content as described below.

### Differentiation of mESCs Into Cardiomyocytes

mESCs were digested with trypsin and resuspended in mESC differentiation medium containing the following: DMEM supplemented with 10% FBS, 0.1 mM non-essential amino acids, 1 mM sodium pyruvate, 0.1 mM β-mercaptoethanol, 100 U/ml penicillin, 100 μg/ml streptomycin and 50 μg/ml ascorbic acid (Sigma-Aldrich, Merck KGaA, Darmstadt, Germany). The cell suspension was seeded into gelatin-coated (0.1%) culture dishes at the density of 5 × 10^6^ cells and left to rest for 20 min to remove the irradiated MEFs; the non-adherent cells were then collected and adjusted to 40 cells/μl in mESC differentiation medium. In order to prepare suspended droplets, the cells were transferred into a 50 ml sterile container, and 25 μl homogenous cell suspension was dropped onto the lid of a bacterial culture dish (150 mm) using a multi-channel pipette. A2Lox-Cre mESCs were then incubated at 37°C (5% CO_2_) to differentiate into embryonic bodies (EBs) via the hanging drop method (Han et al., 2013); each drop contained an average of 1,000 aggregated cells. After 4 days, the EBs were harvested from the droplets and seeded into a gelatin-coated petri dish (0.1%) for further differentiation.

### Quantitative Polymerase Chain Reaction (qPCR)

Total RNA was extracted from the cells or tissues using TRIzol^®^ reagent (Life Technologies, CA, United States) according to the manufacturer’s instructions. The purity and concentration of the RNA were determined using an ultraviolet spectrophotometer, and an A260/A280 ratio in the range of 1.8–2.0 was considered to indicate acceptable purity. 1 μg total RNA was reverse transcribed into cDNA at 37°C for 15 min, 85°C for 5 s, and then stored at 4°C for 30 min. The cDNA was subsequently used as the template for qPCR, which was conducted using the ABI7500 Real-Time PCR System (Applied Biosystems, Thermo Fisher Scientific, Inc.) with GAPDH as the internal reference. The thermocycling conditions were as follows: pre-denaturation at 95°C for 2 min; denaturation at 95°C for 15 s; annealing at 60°C for 15 s; all for 40 cycles. Each sample was run in triplicate and relative expression of the target genes was calculated using the 2^–ΔΔCt^ method (Livak and Schmittgen, 2001). The sequences of all primers are represented in Table 1.

**TABLE 1:** Primer sequences for qPCR.

Gene	Forward sequence	Reverse sequence
*mLHPP*	5′-GACATCTCCGGGGTGCT ATG-3′	5′-CTTTCAGCGGGGACTGTT TCA-3′
*hLHPP*	5′-GAGGCTGGGATTTGACAT CTC-3′	5′-GAGCAGGTATGGTCGC AGG-3′
*mOct4*	5′-GTTGGAGAAGGTGGAAC CAA-3′	5′-CTCCTTCTGCAGGGCT TTC-3′
*mAfp*	5′-CTTCCCTCATCCTCCTGC TAC-3′	5′-ACAAACTGGGTAAAGGTGA TGG-3′
*mMesp1*	5′-ACCCATCGTTCCTGTA CGC-3′	5′-GCATGTCGCTGCTGAA GAG-3′
*mNestin*	5′-CCCTGAAGTCGAGGAG CTG-3′	5′-CTGCTGCACCTCTAAG CGA-3′
*mCDK4*	5′-ATGGCTGCCACTCGATAT GAA-3′	5′-TCCTCCATTAGGAACTCTCA CAC-3′
*mCyclinD1*	5′-GCGTACCCTGACACCAAT CTC-3′	5′-ACTTGAAGTAAGATACGGAG GGC-3′
*mP21*	5′-CCTGGTGATGTCCGA CCTG-3′	5′-CCATGAGCGCATCGCA ATC-3′
*mGAPDH*	5′-CAATCTGTCCGTCGTGG ATC-3′	5′-CCTGCTTCACCACCTTC TTG-3′
*mLefty1*	5′-ACTCAGTATGTGGCCCTG CTA-3′	5′-AACCTGCCTGCCACC TCT-3′
*mTBra*	5′-CTCCAACCTATGCGGAC AAT-3′	5′-CCCCTTCATACATCGGA GAA-3′
*mSox1*	5′-GCGCCCTCGGATCTCTG GTC-3′	5′-GCCCGCGCCCTGGTA GTG-3′
*mSox17*	5′-GGGGCCCATGTGCGGA GAC-3′	5′-GCGGCGCAAGCAGGTG AAG-3′
*mP27*	5′-TCAAACGTGAGAGTGTCTA ACG-3′	5′-CCGGGCCGAAGAGATTT CTG-3′
*m*β*-catenin*	5′-CCCAGTCCTTCACGCAA GAG-3′	5′-CATCTAGCGTCTCAGGGA ACA-3′

*m, mouse; h, human.*

### Western Blotting Analysis

Standard western blot assay: Briefly, the total protein was extracted from cells using RIPA buffer (Solarbio, Beijing, China) on ice, and protein concentration was determined via the BCA method. Following denaturation, the proteins (30 μg of protein loaded per lane) were separated by 10% SDS-PAGE, and then transferred onto PVDF membranes. The membranes were blocked with 5% non-fat dried milk for 2 h, washed 3 times with TBST, and incubated overnight at 4°C with the following primary antibodies: anti-β-actin (dilution, 1:4000; Catalog No. BM0627; Boster Biological Technology), anti-LHPP (dilution, 1:1000; Catalog No. sc-376648, Santa Cruz Biotechnology, Inc.), anti-β-catenin (6B3) Rabbit Monoclonal Antibody (dilution, 1:1000; #9582, Cell Signaling Technology), anti-CDK4 Mouse Monoclonal Antibody (dilution, 1:1000; Catalog No. 66950-1-Ig; Eenchmark Proteintech Group, Wuhan, China), Anti-p21 antibody (dilution, 1:1000; Catalog No. ab188224; Abcam), Anti-p27 Antibody (dilution, 1:1000; Catalog No. ab92741; Abcam), anti-CyclinD1 Mouse Monoclonal Antibody (dilution, 1:1000; Catalog No. 60186-1-Ig; Eenchmark Proteintech, Wuhan, China). The membranes were washed with TBST (5 min × 10 min) and subsequently incubated with Goat Anti-Mouse IgG (H + L) horseradish peroxidase-conjugated Secondary Antibody (dilutions 1:5000; Catalog No. BM3895; Boster Biological Technology) or Goat Anti-Rabbit IgG (H + L) horseradish peroxidase-conjugated Secondary Antibody (dilutions 1:5000; Catalog No. BA1054; Boster Biological Technology) for 3 h at room temperature. Following further washing with TBST (5 min × 10 min), the bands were visualized using an enhanced chemiluminescence detection kit (Advansta, Menlo Park, CA, United States). Quantity One Version 4.6.7 (Bio-Rad Laboratories, Inc., Hercules, CA, United States) was used to quantify the gray values of bands.

Detection of histidine phosphorylation: All procedures were conducted at 4°C, and all reagents were pre-cooled before use. The same number of cells was directly lysed in 2× pHis buffer (250 mM Tris-HCl at pH 8.8, 0.02% bromophenol blue, 50% glycerol, 50 mM EDTA, 500 mM DTT, and 10% SDS) on ice, followed by ultrasonication (3 times for 15 s each). The sediment was removed by centrifugation (10,000 × *g* for 15 min, 4°C). The supernatant was immediately separated at 4°C by modified SDS-PAGE (using a modified stacking gel at pH 7.2, and 10% separation gel at pH 8.8), and then transferred onto PVDF membranes. The membranes were blocked in 2.5% BSA buffer (pH 8.8) (Solarbio, Beijing, China) and then incubated overnight at 4°C with the following primary antibodies: anti-N3-Phosphohistidine (3-pHis) Monoclonal Antibody (Catalog No. MABS1352; Merk KGaA, Germany) and anti-N1-Phosphohistidine (1-pHis) Monoclonal Antibody (Catalog No. MABS1330; Merk KGaA, Germany), diluted in BSA buffer (0.1% Tween-20) to 0.5 μg/ml. The membranes were washed three times (at 4°C for 10 min) with TBST (pH8.8) and incubated with an anti-rabbit HRP-conjugated secondary antibody (dilutions 1:5000) at 4°C. As the control, cell lysates were heated at 100°C for 20 min in loading buffer (pH 8.8) to remove phosphohistidine. Lanes loaded with the heated lysates indicated either heat-resistant phosphohistidine or non-specific signals. Absolute gray value of intracellular phosphohistidine = gray value of samples - gray value of the control sample. Relative gray value = absolute gray value/gray value of reference gene.

### Immunofluorescence Assay

Firstly, sterile slides were placed into each well of a 6-well plate. Cells in the logarithmic growth phase were digested with 0.25% trypsin, and then seeded into the plates at a density of 1 × 10^5^ cells per well. The following day, the slides were washed 3 times with PBS, and then fixed with 4% paraformaldehyde for 30 min at room temperature prior to additional washing as aforementioned. The slides were then treated with 0.25% Triton for 10 min, and washed again prior to blocking with 5% BSA solution for 1 h. Following overnight incubation with the primary antibodies: anti-Oct3/4 (C-10) Mouse monoclonal Antibody (dilution, 1:300; Catalog No. sc-5279; Santa Cruz Biotechnology, Inc.) and anti-Lefty (F-11) Mouse monoclonal Antibody (dilution, 1:300; Catalog No. sc-166708; Santa Cruz Biotechnology, Inc.) at 4°C, the slides were washed again with PBS (3 times), and incubated with Goat anti-Mouse IgG2b Cross-Adsorbed Secondary Antibody, Alexa Fluor 555 (dilution, 1:300; Catalog No. A-21147; Life Technology) and Goat anti-Rabbit IgG (H + L) Cross-Adsorbed Secondary Antibody (dilution, 1:300; Catalog No. A-21428; Life Technology) at room temperature for 3 h. The slides were then washed with PBS 3 more times, counterstained with DAPI solution (dilution, 1:10,000; Catalog No. C0060; Solarbio, Beijing, China) for 5 min and washed with PBS a further 3 times. Finally, the slides were sealed with 50% glycerol and observed under a fluorescence microscope (Olympus Corporation, Tokyo, Japan). Magnification, 100×.

### Cell Cycle Assay

mESCs were collected by centrifugation at 200 × *g* for 3 min at room temperature, and fixed with 70% pre-cooled ethanol at 4°C overnight. The following day, the cells were centrifuged at 700 × *g* for 3 min at room temperature, washed with pre-cooled PBS and resuspended (also in pre-cooled PBS) to a final concentration of 1.0 × 10^6^ cells/ml. RNase A (10 μg/ml) was added to 100 μl cell suspension and the samples were harvested by centrifugation (as aforementioned). The cells were washed with pre-cooled PBS, resuspended and filtered using a 75 μm nylon mesh; Cell cycle analysis was detected using a flow cytometer (Guava EasyCyte 8, Merk), and the data were analyzed using FlowJo 7.6 Software (FlowJo LLC, Ashland, OR, United States).

### Cell Proliferation Assay

mESCs in the logarithmic growth phase were digested with trypsin, resuspended in culture medium, and then seeded into 96-well plates at a density of 1 × 10^4^ cells per well. 10% of Cell Counting Kit-8 (CCK-8) reagent (Cat. C0038, Beyotime Biotechnology, China) was added at 12, 24, 36, 48, 60, and 72 h time points, followed by incubation at 37°C for 2 h. Absorbance values were detected with a microplate reader (Bio-Rad) at 450 nm.

### Alkaline Phosphatase (AP) Staining

Stem cell properties were validated by AP staining. The cells treated with RA were used as a positive control, while untreated cells (receiving neither RA nor DOX) were used as a negative control. Briefly, mESCs were fixed with 4% formaldehyde for 2 min at room temperature. The cells were subsequently stained using the Alkaline Phosphatase Kit (SiDanSai Biotechnology, Shanghai, China) according to the manufacturer’s instructions. After washing 3 times with PBS, the cells were observed under an optical microscope (Leica DM750). Magnification, 100×.

### Colony Formation Assay

mRSCs were inoculated into 6-well plates at a density of 500 cells per well and incubated for 10 consecutive days. Culture medium was changed every 2 days. Clones were observed, and the culture was terminated when the clones grew to the naked eye. After fixation with 75% ethanol for 10 min at room temperature, the cells were stained with 0.1% crystal violet solution for 15 min. Images were photographed under a microscope (Leica DM750).

### Bromodeoxyuridine (Brdu) Staining

For BrdU staining, when mESCs grown to 60% confluence, Brdu solution (10 μg/ml, Sigma) was added into culture medium. After 6 h of incubation, the cells were collected and fixed with 75% ethanol for 30 min at room temperature. Next, the cells were treated with 1 nM HCl for 30 min. After discarding the HCl, the cells were rinsed with 10 μM boric acid, treated with 0.1% Triton for 30 min, blocked with 5% BSA for 1 h, incubated with anti-BrdU primary antibody (ab6326, 1:200 dilution, Abcam) diluted with 5% BSA for 12 h at 4°C, and then labeled with corresponding fluorophore-conjugated secondary antibody (#4416, 1:400 dilution, Cell Signaling Technology) for 2 h at room temperature. Flow cytometric data were analyzed using FlowJo 7.6 Software (FlowJo LLC, Ashland, OR, United States).

### Animal Experiments

C57BL/6 mice (*n* = 10), aged 6–8 weeks and weighing 18.5 ± 1.9 g each, were purchased from the animal center of Xiamen University. The mice were euthanized by cervical dislocation. Adult mouse tissues, including muscle tissue, brain tissue, kidney, gut, spleen, bladder, lung, and liver were carefully excised and kept in liquid nitrogen immediately. All animal experiments were conducted with the approval of the animal ethics and use committee (IACUC) of Xiamen university (Xiamen, Fujian, China; Approval ID: scxk2013-0006).

### Statistical Analysis

SPSS 21.0 (IBM Corporation, Armonk, NY, United States) and GraphPad Prism 6.0 (GraphPad Software, Inc., La Jolla, CA, United States) were used for data analysis and graphical representation, respectively. The difference between two groups was determined using the Student’s *t*-test, and comparisons among multiple groups were measured using one-way ANOVA with Dunnett’s test. Data were expressed as mean ± Standard Deviation (SD). Each experiment was independently repeated three times, and *P* < 0.05 was considered as statistically significant.

## Results

### *Lhpp* Expression Is Up-Regulated During mESCs Differentiation

**FIGURE 1:**
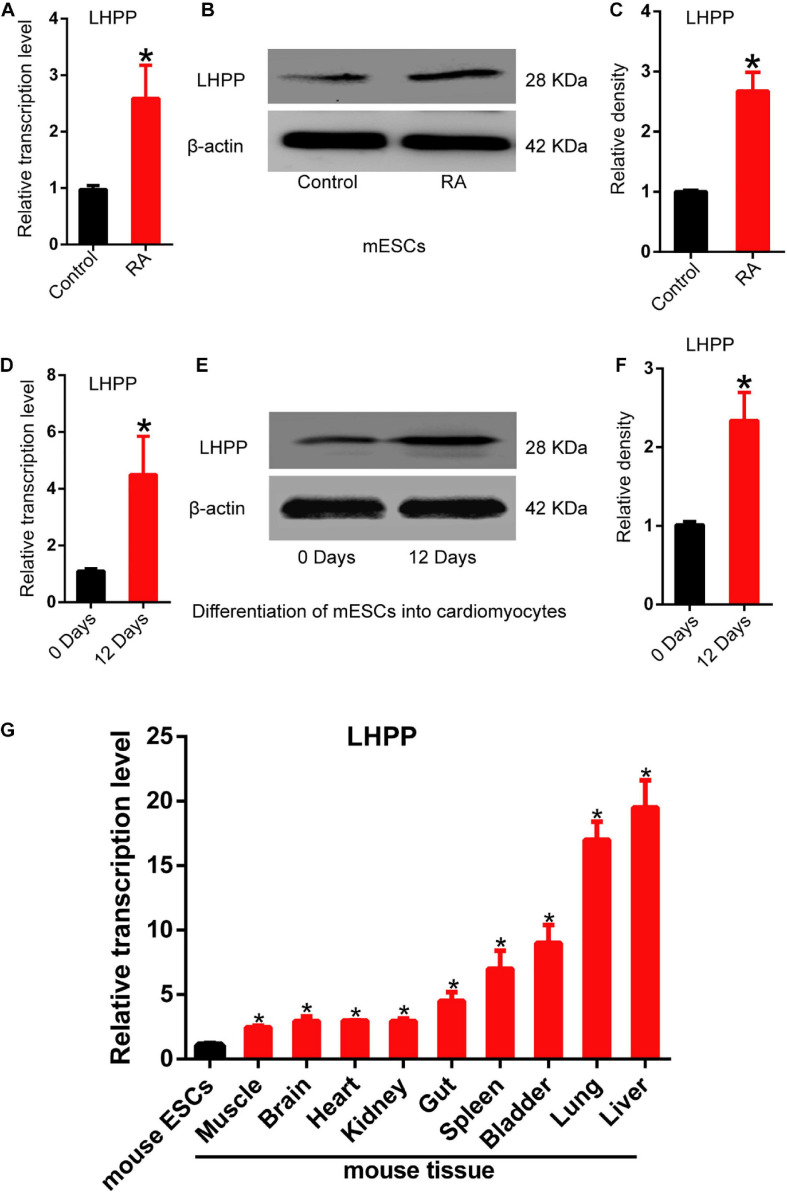
Up-regulation of *Lhpp* expression during mES cell differentiation. **(A–C)** Up-regulated expression of m*Lhpp* in mESCs treated with 10 mM of RA for 3 days. **(A)** Expression of m*Lhpp* mRNA; **(B)** representative blots of mLHPP expression; **(C)** statistical analysis of the protein expression of mLHPP. **(D–F)** Increased expression of m*Lhpp* in mESCs during myocardial differentiation, as validated by qPCR and western blotting analysis. **(D)** Statistical results of qPCR; **(E)** representative blots; **(F)** quantification of protein expression. **(G)** Up-regulated transcription of m*Lhpp* in adult mouse tissues, compared with mESCs. GAPDH was used as the internal control. All experiments were independently repeated at least three times, and the data are presented as the mean ± SD. *P < 0.05. LHPP, phospholysine phosphohistidine inorganic pyrophosphate phosphatase; m*Lhpp*, mouse *Lhpp*; mESCs, mouse embryonic stem cells; RA, retinoic acid.

To understand the role of LHPP in mouse ESCs differentiation, differentiation of A2Lox-Cre mESCs to cardiomyocytes was induced via embryoid bodies (EBs). We observed a weakened self-renewal ability and loss of stemness in mESCs after RA treatment for 72 h, in which *Lhpp* expression was significant upregulated (Figures 1A–C). Besides, the expression of *Lhpp* was increased during myocardial differentiation of mESCs at transcriptional and translational levels (Figures 1D–F). Furthermore, data indicated that *Lhpp* was more highly expressed in adult mouse tissues than in mESCs (Figure 1G).

### Successful Construction of DOX-Induced *Lhpp*-Overexpressing and *Lhpp*-Silenced mESC Lines

**FIGURE 2:**
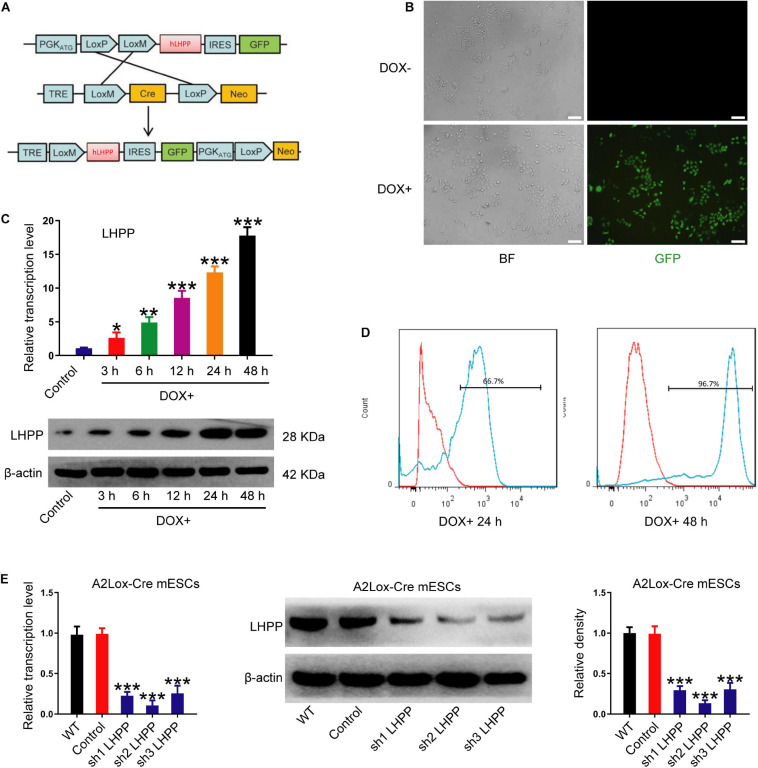
Construction and identification of DOX-induced h*Lhpp*-overexpressing and -silenced mESCs. **(A)** Schematic diagram depicting the production of DOX-induced hLHPP-overexpressing mESCs. **(B)** Fluorescence detection of the mESCs following DOX treatment (1 μg/ml) for 48 h. Scale bar = 100 μm. **(C)** The mRNA and protein expression levels of *Lhpp* following DOX treatment (1 μg/ml) at different time points. **(D)** GFP expression in mESCs following DOX treatment (1 μg/ml) for 24 or 48 h was detected by flow cytometry. **(E)** The mRNA and protein expression levels of *Lhpp* were determined by qPCR and western blotting analysis in *Lhpp*-silenced mESCs. All experiments were independently repeated at least three times, and the data are presented as the mean ± SD. *P < 0.05, **P < 0.01, ***P < 0.001. DOX, doxycycline; LHPP, human phospholysine phosphohistidine inorganic pyrophosphate phosphatase; h*Lhpp*, human *Lhpp;* mESCs, mouse embryonic stem cells; RA, retinoic acid.

To explore the role of LHPP in mESCs, DOX-induced hLHPP-overexpressing mESC line was constructed via homologous recombination (Figure 2A). Following DOX treatment, immunofluorescence, qPCR, and western blotting revealed that the successful construction of human *Lhpp* (h*Lhpp*)-overexpressing mESC line, in which the expression of DOX-induced h*Lhpp* was up-regulated, compared with that in the untreated cells at different time points (Figures 2B,C); meanwhile, flow cytometry analysis suggested that DOX induction influenced the fluorescence intensity of GFP in a time dependent manner (Figure 2D). To determine the biological function of endogenous LHPP in mESCs, we constructed three shRNAs targeting mouse *Lhpp* and the silencing efficiency of these 3 shRNAs in A2Lox-Cre ESCs cells was verified. sh2 RNA with the highest silencing efficiency was selected for subsequent experiments (Figure 2E). These results confirmed the successful construction of DOX-induced *Lhpp*-overexpressing mESCs and *Lhpp* knockdown mESCs.

### LHPP-Mediated Histidine Dephosphorylation Regulates the Expression of Pluripotent- and Differentiation-Related Genes in mESCs

**FIGURE 3:**
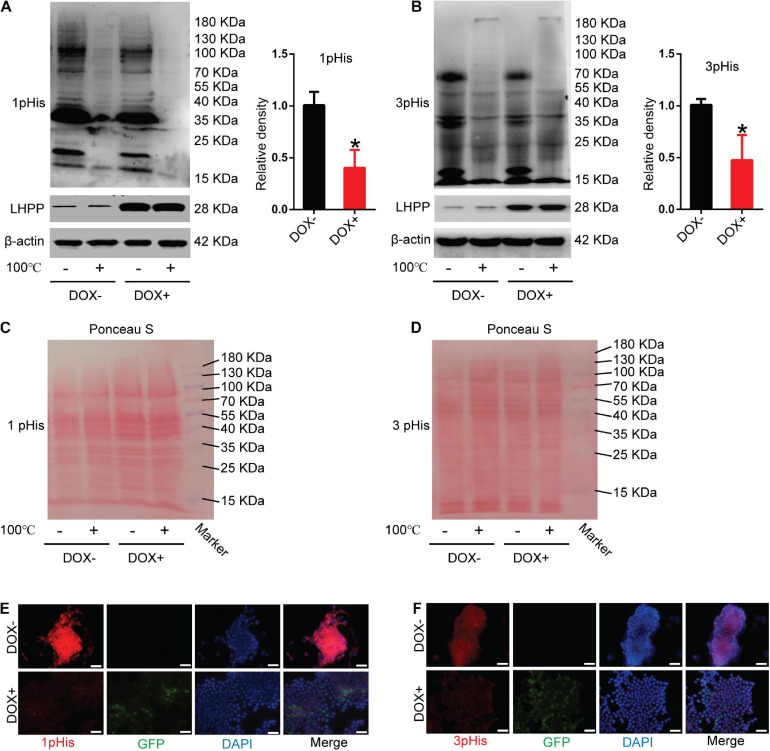
Effect of human LHPP-overexpression on histidine phosphorylation in mESCs. **(A)** Intracellular 1-pHis levels were detected by modified western blotting in mESCs treated with 1 μg/ml DOX for 72 h. **(B)** Intracellular 3-pHis levels in mESCs were detected by modified western blotting following treatment with 1 μg/ml DOX for 72 h. **(C,D)** Ponceau’s staining was used to validate the amount of protein loaded per well. **(E,F)** Immunofluorescence was carried out to observe the **(E)** 1-pHis and **(F)** 3-pHis levels in mESCs treated with 1 μg/ml DOX for 72 h. Red, 1-pHis or 3-pHis; green, GFP; blue, DAPI. Scale bar = 100 μm. All experiments were independently repeated at least three times, and the data are presented as the mean ± SD. *P < 0.05. LHPP, human phospholysine phosphohistidine inorganic pyrophosphate phosphatase; mESCs, mouse embryonic stem cells; DOX, doxycycline.

To unveil the mechanism by which LHPP regulates the biological behavior of mESCs, the level of intracellular histidine phosphorylation was measured using modified SDS-PAGE and immunofluorescence assay. As illustrated in Figures 3A,B, the levels of 1-pHis and 3-pHis were found to be reduced in mESCs overexpressing LHPP, compared with those not induced with DOX. Protein samples that were pretreated at 100°C for 20 min exhibited non-specific bands, which may have been due to the low specificity of the antibody, or an excessive incubation time. For loading control, Ponceau’s staining was conducted (Figures 3C,D). Besides, similar results of 1-pHis and 3-pHis levels were obtained using immunofluorescence (Figures 3E,F).

**FIGURE 4:**
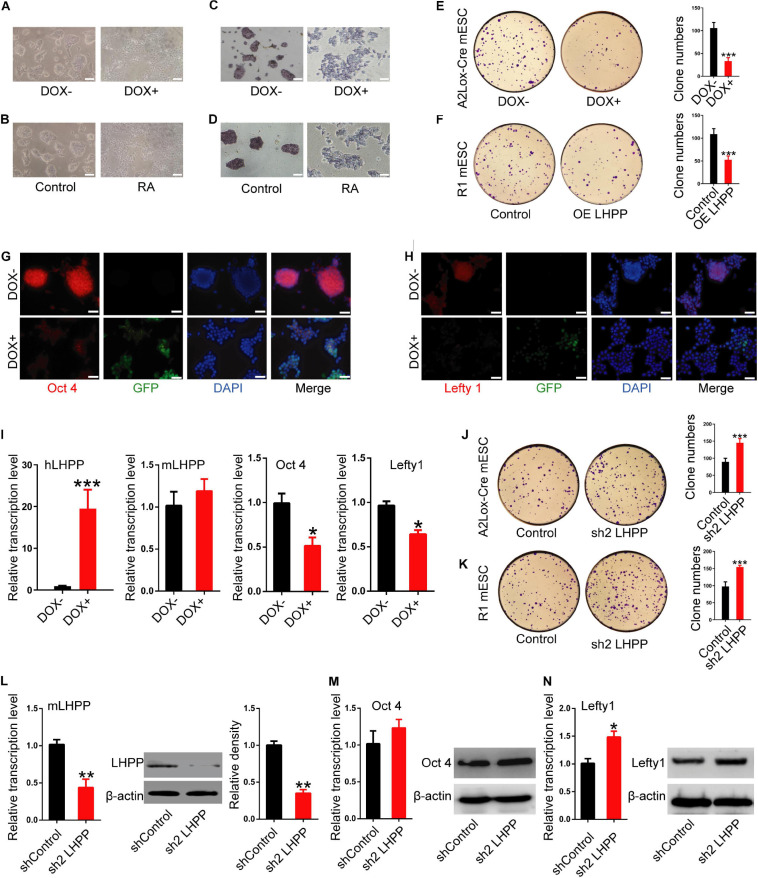
Effects of LHPP on the expression of cellular pluripotency and differentiation-related genes in mESCs. **(A)** mES cell colonies were observed with or without DOX treatment (1 μg/ml) for 72 h. **(B)** mES cell colonies after incubation with or without 10 mM RA for 72 h. **(C,D)** AP staining was performed in mESCs with or without **(C)** DOX (1 μg/ml) or **(D)** 10 mM RA treatment for 72 h. **(E,F)** Colony-formation assay was performed to detected the effects of *Lhpp* overexpression on **(E)** A2Lox-Cre mESCs and **(F)** R1 mESCs. **(G,H)** Immunofluorescent labeling of **(G)** Oct4 and **(H)** Lefty1 in DOX-induced h*Lhpp*-overexpressing mESCs. Red, Oct4 or Lefty1; green, GFP; blue, DAPI. Scale bar = 100 μm. **(I)** qPCR quantification of the mRNA levels of human *Lhpp*, mouse *Lhpp*, *Oct4* and *Lefty1* in mESCs treated with or without DOX (1 μg/ml). **(J,K)** Colony-formation assay was performed to evaluate the effects of *Lhpp* knockdown on **(J)** A2Lox-Cre mESCs and **(K)** R1 mESCs. **(L)** qPCR detection of m*Lhpp* expression in mESCs. **(M,N)** The mRNA and protein levels of **(L)** *Oct4* and **(M)** *Lefty1* in Lhpp-silenced mESCs. All experiments were independently repeated at least three times, and the data are represented as the mean ± SD. *P < 0.05, **P < 0.01, ***P < 0.001. DOX, doxycycline; LHPP, human phospholysine phosphohistidine inorganic pyrophosphate phosphatase; h*Lhpp*, human *Lhpp*; mESCs, mouse embryonic stem cells; RA, retinoic acid; AP, alkaline phosphatase; sh, short hairpin RNA.

To further understand the role of LHPP in mESC self-renewal, stem cell proprieties were validated by AP staining. Seventy-two hours after DOX induction (Figure 4A) or RA treatment (Figure 4B), the cell colonies were decentralized with irregular edges, and AP staining was reduced compared with the control (Figures 4C,D). Colony formation experiments further confirmed that the clonogenic capacity of both DOX-induced A2Lox-Cre mESCs and R1 mESCs overexpressing LHPP by lentiviral infection was significantly reduced (Figures 4E,F). Immunofluorescence, qPCR and western blotting analysis revealed that the expression levels of stem cell marker genes *Oct4* and *Lefty1* were significantly down-regulated (Figures 4G–I and Supplementary Figures 1A–C, 2A) in mESCs with *Lhpp* overexpression compared with the control cells, where there was no significant change in endogenous mouse *Lhpp* expression following the exogenous overexpression of human *Lhpp*. Meanwhile, the mRNA levels of *T-Bra*, *Mesp1*, *Nestin*, *SOX1*, *SOX17*, *Afp* were significantly elevated in LHPP-overexpressed mESCs (Supplementary Figure 1D). Conversely, silencing of *Lhpp* in A2Lox-Cre mESCs and R1 mESCs enhanced the clonogenic ability of mESCs (Figures 4J,K). Besides, *Lhpp* knockdown upregulated *Lefty1* expression at the mRNA and protein levels, but reduced the mRNA levels of *T-Bra*, *Mesp1*, *Nestin*, *SOX1*, *SOX17*, *Afp* in mESCs as compared to the control group (Figures 4L–N and Supplementary Figures 1E,F, 2B). No significant difference in Oct4 expression was observed in DOX-induced LHPP overexpressing mESCs, while *Lhpp* knockdown by shRNA upregulated *Oct 4* mRNA expression in R1 mESCs compared to the control. Overall, these findings suggested that LHPP plays role in differentiation of mESCs.

### The Enzymatic Active Site of LHPP Is the Cysteine Residue at Position 226, Not 53, Which Is Critical for mESCs Self-Renewal

**FIGURE 5:**
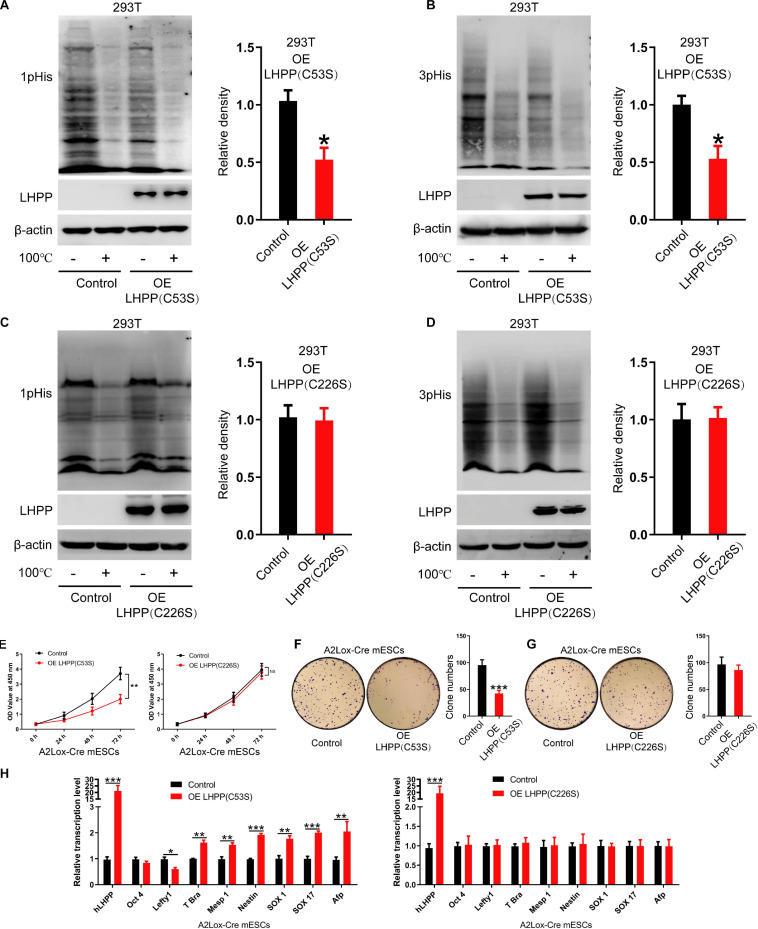
The cysteine at position 226 of LHPP is essential for its histidine phosphatase activity. **(A,B)** Western blotting analysis for intracellular **(A)** 1-pHis and **(B)** 3-pHis levels after overexpression of LHPP (C53S) in 293T cells. **(C,D)** Western blotting analysis for intracellular **(C)** 1-pHis and **(D)** 3-pHis levels after overexpression of LHPP (C226S) in 293T cells. **(E)** Cell proliferative ability was detected by CCK-8 assay after overexpression of LHPP (C53S) mutant or LHPP (C226S) mutant in A2Lox-Cre mESCs. **(F,G)** Cell colony formation capability was assayed by colony formation assay after overexpression of **(F)** LHPP (C53S) mutant or **(G)** LHPP (C226S) mutant in A2Lox-Cre mESCs. **(H)** After overexpression of LHPP (C53S) mutant or LHPP (C226S) mutant in A2Lox-Cre mESCs, the expression levels of pluripotency and differentiation-associated genes were evaluated by qPCR analysis. All experiments were independently repeated at least three times, and the data are represented as the mean ± SD. *P < 0.05, **P < 0.01, ***P < 0.001. NS, not significant difference. LHPP, phospholysine phosphohistidine inorganic pyrophosphate phosphatase.

Although previous literature has speculated that the specific enzymatic active site of LHPP is the cysteine residues at position 53 and 226 (Yokoi et al., 2003), so far, no study has confirmed this. In present study, we constructed two mutant plasmids of *Lhpp* as described in the methods, resulting in mutant proteins LHPP (C53S) and LHPP (C226S) (Supplementary Figures 3A,B). After overexpression of these two mutants in human 293T cells, respectively, we detected that the LHPP (C53S) mutant was able to significantly reduce intracellular histidine phosphorylation, but the LHPP (C226S) mutant did not produce this effect (Figures 5A–D).

To determine the effects of overexpression of these two catalytic mutants induced by DOX on the self-renewal of mESCs, CCK-8 assay, qPCR analysis for pluripotency and differentiation markers and colony formation assay was performed in A2Lox-Cre mESCs. We found that overexpression of LHPP (C53S) mutant significantly attenuated cell proliferative ability, while there was no significant difference between LHPP (C226S) mutant-overexpressed mESCs and the control group (Figure 5E). Besides, colony formation assay confirmed that overexpression of LHPP (C53S) mutant resulted in significantly reduced colony formation capability in mESCs; however, mutating LHPP (C226S) had no significant effect (Figures 5F,G). Meanwhile, qPCR analysis indicated that both mutants were successfully overexpressed in the mESCs. Similar to the aforementioned results, LHPP (C53S) mutant markedly decreased the mRNA expression of pluripotency gene *Lefty1*, but elevated the expression of differentiation-associated genes *T-box transcription factor Brachyury* (*T-Bra*), *Mesp1*, *Nestin*, *SOX1*, *SOX17*, and *Afp* (Figure 5H). In contrast, although LHPP (C226S) mutant was also significantly upregulated in mESCs, it did not produce similar results. Cumulatively, these data suggest that the enzymatic active site of LHPP is the cysteine residue at position 226, which is essential for maintaining its function to regulate mESCs self-renewal.

### LHPP-Mediated Histidine Dephosphorylation Modulates the Proliferation of mESCs by Cell Cycle Arrest

**FIGURE 6:**
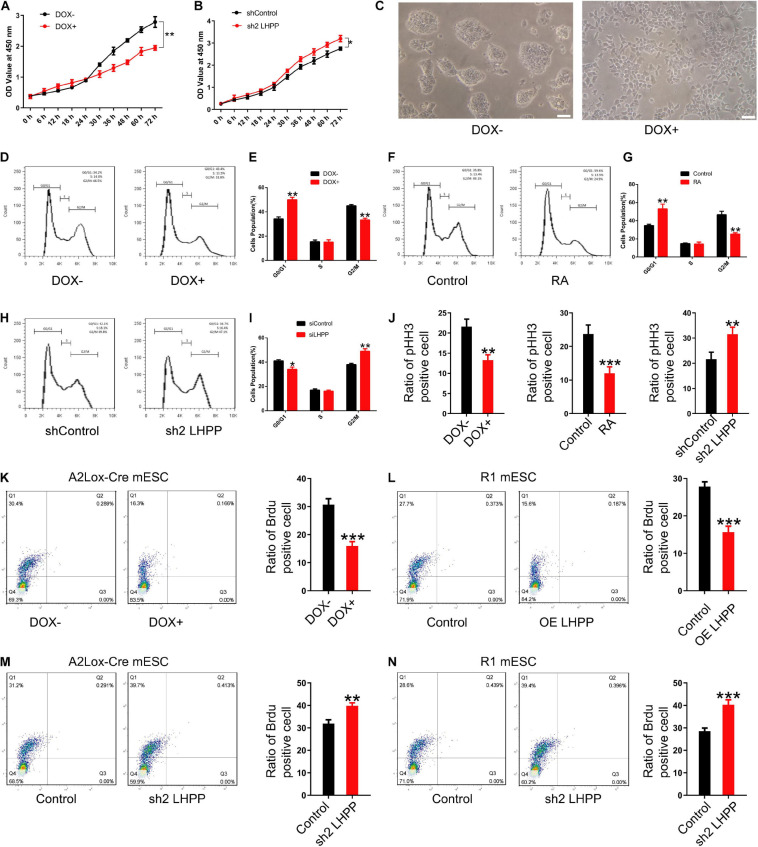
Effect of LHPP on mESC proliferation and cell cycle. **(A,B)** The Cell Counting Kit-8 assay was used to detect the proliferation of **(A)** mESCs following treatment with DOX (1 μg/ml) and **(B)** shRNA targeting Lhpp. **(C)** mES cell colonies following DOX treatment (1 μg/ml) for 72 h. Scale bar = 100 μm. **(D,E)** Flow cytometry was conducted to detect changes to the cell cycle in mESCs treated with DOX (1 μg/ml) for 3 days. **(E)** Quantification. **(F,G)** RA-treated (10 mM) cells were used as the positive control. Cells without DOX treatment (1 μg/ml) were used as the negative control group. **(G)** Quantification. **(H,I)** Flow cytometry was used to determine the changes in the cell cycle in *Lhpp* knockdown mESCs cultured for 3 days, respectively. **(I)** Quantification. **(J)** The percentage of pHH3-positive cells in mESCs. **(K,L)** The percentage of Brdu-positive cells in **(K)** DOX-induced (1 μg/ml) LHPP overexpressing mESCs and **(L)** R1 mESCs with LHPP overexpression. **(M,N)** The percentage of Brdu-positive cells in *Lhpp* knockdown **(M)** A2Lox-Cre mESCs and **(N)** R1 mESCs. All experiments were independently repeated at least three times, and the data are presented as the mean ± SD. *P < 0.05, **P < 0.01, ***P < 0.001. LHPP, phospholysine phosphohistidine inorganic pyrophosphate phosphatase; mESCs, mouse embryonic stem cells; DOX, doxycycline; RA, retinoic acid; shRNA, short hairpin RNA.

To investigate how LHPP exerts its biological effects, flow cytometry was performed to detect changes of cell-cycle progression. It was revealed that when cultured with medium containing LIF, the proliferative capacity of DOX-induced LHPP-overexpressing mESCs was dramatically attenuated (Figures 6A,C), and that the proliferation of mESCs slowed down, compared with cells of the control group (Figures 6D,E), which was consistent with what was observed in RA treated (RA+) group (Figures 6F,G). Conversely, proliferation of *Lhpp* knockdown mESCs was significantly enhanced compared with that in the control cells (Figure 6B). Moreover, compared with wild-type cells, *Lhpp* knockdown mESCs cultured in LIF medium showed significant changes in cell cycle distribution, with an increase in the proportion of G_2_/M cells, but a decrease in the proportion of G_0_/G_1_ cells (Figures 6H,I). In addition, DOX-induced overexpression of LHPP exhibits a lower proportion of pHH3-positive cells than the control cells. These results were consistent with the results from RA-induced ESCs differentiation. Conversely, *Lhpp* knockdown caused an increased proportion of pHH3-positive cells (Figure 6J). Further, Brdu staining showed that overexpression of LHPP in A2Lox-Cre mESCs and R1 mESCs significantly reduced the percentage of Brdu-positive cells, whereas silencing of *Lhpp* in both cells yielded opposing effects (Figures 6K–N).

We further repeated the above experiments with LIF-free culture medium. Data indicated that the proliferation and cell cycle of mESCs were affected after culturing with LIF-free medium for 3 days, in comparison to cells treated with medium containing LIF; besides, when the cells were cultured in LIF-free medium, the inhibitory effect of DOX-induced LHPP-overexpression on the cell cycle was enhanced, resulting in a slower proliferation of mESCs (Supplementary Figures 4A,C,D). Nevertheless, the proliferation of *Lhpp* knockdown mESCs cultured in LIF-free medium were promoted (Supplementary Figure 4B) and cell cycle was changed significantly as in LIF medium (Supplementary Figures 4E,F). Collectively, our findings suggest that LHPP suppresses the proliferation of mESCs and induces cell cycle arrest, and that these effects are enhanced in the absence of LIF treatment.

### LHPP-Mediated Histidine Dephosphorylation Regulates the Expression of Wnt Signaling Pathway-Related Molecules and Cell Cycle-Related Genes

**FIGURE 7:**
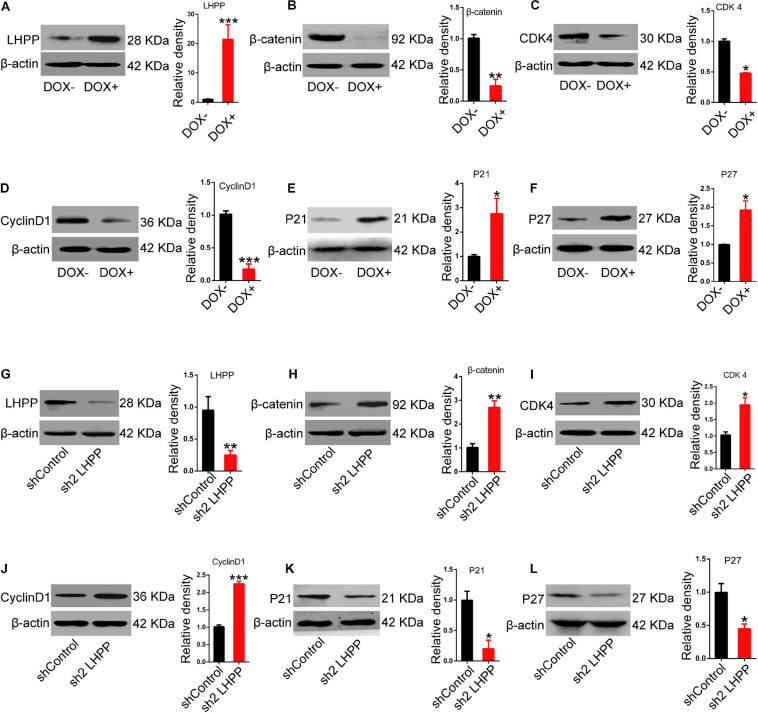
Effect of LHPP-overexpression on the expression levels of Wnt signaling pathway-related molecules and cell cycle-related genes. **(A–F)** The protein expression levels of **(A)** hLHPP, **(B)** β-catenin, **(C)** CDK4, **(D)** CyclinD1, **(E)** P21, and **(F)** P27 in A2Lox-Cre mESCs treated with DOX (1 μg/ml) for 72 h were detected by western blotting. **(G–L)** The protein expression levels of **(G)** mLHPP, **(H)** β-catenin, **(I)** CDK4, **(J)** CyclinD1, **(K)** P21, and **(L)** P27 in Lhpp-silenced A2Lox-Cre mESCs cultured for 72 h. All experiments were independently repeated at least three times, and the data are presented as the mean ± SD. *P < 0.05, **P < 0.01, ***P < 0.001. LHPP, phospholysine phosphohistidine inorganic pyrophosphate phosphatase; hLHPP, human LHPP; mLHPP, mouse LHPP; mESCs, mouse embryonic stem cells; DOX, doxycycline; shRNA, short hairpin RNA.

Mechanistically, we detected the expression of Wnt signaling pathway-related molecules and cell cycle-related genes. Data showed that the mRNA (Supplementary Figure 5A) and protein (Figure 7) expression levels of β*-catenin*, and the cell cycle-promoting genes *CDK4* and *CyclinD1* were significantly attenuated, whilst those of the cell cycle-inhibitory genes *P21* and *P27* were markedly increased in DOX-induced LHPP-overexpressing mESCs, in comparison to the control cells (when both were cultivated with medium containing LIF). By contrast, *Lhpp* knockdown produced the opposite results (Supplementary Figure 5B).

## Discussion

In this study, our findings suggested that *Lhpp* expression was up-regulated during mouse ESCs differentiation. Via construction of DOX-induced LHPP-overexpressing and LHPP-silenced mouse ESC lines, we further found that *Lhpp* overexpression contributed to the down-regulation of histidine phosphorylation. Importantly, data indicated that the enzymatic active site of LHPP is the cysteine residue at position 226, not 53. Moreover, LHPP-mediated histidine dephosphorylation modulates the proliferation of mESCs by cell cycle arrest, where the expression of pluripotent- and differentiation-related genes, Wnt signaling pathway-related molecules and cell cycle-related genes were altered as well. Overall, our data provide new insight into how LHPP influences ESCs self-renewal.

Embryonic stem cells are capable of self-renewal and have the potential to differentiate into a variety of different cell types. Their proliferation (self-renewal) and differentiation are precisely regulated at the transcriptional, translational and post-translational levels in a state of dynamic equilibrium (Si et al., 2016; Liou et al., 2017; Ran et al., 2017). Mounting evidences have revealed that post-transcriptional modification plays a vital role in ESCs self-renewal and differentiation. However, the exact molecular mechanisms remain poorly studied. Previous studies have shown that post-translational modifications, such as protein ubiquitination and phosphorylation, are important components of the ESCs self-renewal regulatory network. For protein phosphorylation, most studies have focused on the phosphorylation of serine, threonine and tyrosine residues. However, histidine can also undergo phosphorylation modification. LHPP was identified as a protein histidine phosphatase, which may act as a liver cancer suppressor gene (Hindupur et al., 2018). Mechanistically, LHPP regulates the expression and function of a series of genes via histidine dephosphorylation in cells, thereby inhibiting cellular proliferation (Hindupur et al., 2018; Zheng et al., 2018). Previous studies have reported that LHPP is associated with a variety of neurological diseases (Converge consortium, 2015; Polimanti et al., 2017), and is also implicated in the development of hematological cancers (Gutierrez-Camino et al., 2017; Vijayakrishnan et al., 2017). However, whether LHPP-mediated histidine dephosphorylation can regulate the biological behavior of ESCs remains largely unknown.

In present study, *Lhpp* expression was markedly upregulated after myocardial differentiation of mESCs or mESCs treated with RA, at both the transcriptional and translational levels. However, the role of *Lhpp* expression and LHPP-mediated histidine dephosphorylation in ESCs cells deserve further investigation. Herein, we constructed a DOX-induced human LHPP-overexpression mESCs via homologous recombination, as previously reported (Iacovino et al., 2014; Zhang et al., 2016). An shLHPP ESC line was also constructed. Western blotting and immunofluorescence assays demonstrated that following LHPP-overexpression, the level of histidine phosphorylation in ESCs was significantly downregulated. Furthermore, consistent with the observations in the positive control group (RA treatment group), colonies of ESCs overexpressing LHPP were dispersed and the levels of AP were attenuated, suggesting that LHPP inhibits ESCs proliferation. Furthermore, our data revealed that the expression levels of Oct4 and Lefty1 were significantly downregulated, while the expression levels of genes related to the differentiation were markedly upregulated. By contrast, *Lhpp* silencing was able to reverse the gene expression mentioned above. Our findings indicated that LHPP-mediated histidine dephosphorylation may suppress ESCs self-renewal.

In further experiments, the CCK-8 assay was used to validate the observation that LHPP overexpression significantly inhibited ESCs proliferation, whereas LHPP silencing promoted ESCs proliferation. Cell cycle assays revealed a significant change in the cell cycle distribution of mESCs overexpressing LHPP, and a marked increase in the number of cells in the G_0_/G_1_ phase, which was consistent with the results in ESCs cells treated with RA. However, in *Lhpp* knockdown ESCs, an increased proportion of G_2_/M phase cells was observed.

In a further mechanistic study, it was found that LHPP-mediated histidine dephosphorylation significantly downregulated the mRNA and protein expression levels of *CDK4* and *CyclinD1*, which promote cell cycle progression, whereas the expression of the cell cycle-related inhibitory genes *P21* and *P27* was significantly increased. Although previous studies have identified multiple target proteins of LHPP via mass spectrometry, including essential fatty acid synthetase ATP-citrate lyase (ACLY) (Hindupur et al., 2018), how LHPP exerts its biological function through its substrates, which needed future studies. Interestingly, the expression level of β-catenin was considerably downregulated in our study. β-catenin is a key protein of the Wnt signaling pathway (Clevers and Nusse, 2012; Srivastava et al., 2018) that can trigger the expression of a series of genes, including CyclinD1 and CDK4, which subsequently regulate the expression of genes associated with the cell cycle (Yang et al., 2014; Tao et al., 2017). Wnt signaling pathway is one of the mechanisms affecting stem cell proliferation and differentiation (Xu et al., 2016; Selvaraj et al., 2017). Therefore, we hypothesized that LHPP affects the proliferation and differentiation of ESCs (and potentially human pluripotent stem cells) by regulating Wnt signaling, which warrants further investigation.

## Data Availability Statement

The raw data supporting the conclusions of this article will be made available by the authors, without undue reservation.

## Ethics Statement

All animal experiments were conducted with the approval of the animal ethics and use committee (IACUC) of Xiamen University (Xiamen, Fujian, China; Approval ID: scxk2013-0006).

## Author Contributions

RX: conception and design, collection and/or assembly of data, data analysis and interpretation, and manuscript writing. DY and XC: collection and/or assembly of data. XX: conception and design, financial support, administrative support, manuscript writing, and final approval of manuscript. All authors contributed to the article and approved the submitted version.

## Conflict of Interest

The authors declare that the research was conducted in the absence of any commercial or financial relationships that could be construed as a potential conflict of interest.

## Supplementary Material

Supplementary Figure 1 The effects of LHPP overexpression or silencing on gene expressions. **(A–C)** qPCR and western blotting quantification of **(A)** m*Lhpp*, **(B)** *Oct4*, and **(C)** *Lefty1* levels in A2Lox-Cre mESCs treated with LIF alone or in combination with DOX (1 μg/ml). **(D)** qPCR quantification of the mRNA levels of *SOX17*, *Afp, T-Bra*, *Mesp1*, *Nestin*, and *SOX1* in mESCs treated with LIF alone or in combination with DOX (1 μg/ml). **(E,F)** qPCR detection of the mRNA levels of *SOX17*, *Afp*, *T-Bra*, *Mesp1*, *Nestin* and *SOX1* **(E)** in mESCs transfected with shRNA targeting Lhpp, or **(F)** in A2Lox-Cre mESCs treated with RA (10 mM). All experiments were independently repeated at least three times, and the data are represented as the mean ± SD. ^∗^*P* < 0.05, ***P* < 0.01, ****P* < 0.001. LHPP, phospholysine phosphohistidine inorganic pyrophosphate phosphatase; mLHPP, mouse LHPP; mESCs, mouse embryonic stem cells; DOX, doxycycline; LIF, leukemia inhibitory factor; RA, retinoic acid.

Supplementary Figure 2 The expression of *Lefty1* and *Oct4* in *Lhpp*-overexpressed and silenced R1 mESCs. **(A)** *Lefty1* and *Oct4* mRNA expression were downregulated in *Lhpp*-overexpressed R1 mESCs. **(B)** *Lefty1* and *Oct4* mRNA expression were upregulated in L *Lhpp*-silenced R1 mESCs. All experiments were independently repeated at least three times, and the data are represented as the mean ± SD. **P* < 0.05, ***P* < 0.01. LHPP, phospholysine phosphohistidine inorganic pyrophosphate phosphatase; mESCs, mouse embryonic stem cells.

Supplementary Figure 3 Schematic diagram of the mutation sites in *Lhpp*. **(A)** Sanger sequencing confirmed the successful mutation of LHPP at position 158, labeled as LHPP (c.158G > C). The corresponding amino acid sequence is LHPP (C53S). **(B)** Sanger sequencing showed the successful mutation of LHPP at position 677, labeled LHPP (c.677G > C). The corresponding amino acid sequence is LHPP (C226S). The mutation sites are colored in red.

Supplementary Figure 4 Effect of LHPP on proliferation and cell cycle of mESCs. **(A,B)** Cell Counting Kit-8 assay was used to detect the proliferation of **(A)** A2Lox-Cre mESCs following treatment with DOX (1 μg/ml) alone or in combination with LIF and **(B)** *Lhpp* knockdown mESCs cultured with LIF-free medium. **(C,D)** Flow cytometry was used to detect changes to the cell cycle in mESCs cultured with LIF-free medium, with or without DOX (1 μg/ml) for 3 days, respectively. **(D)** Quantification. **(E,F)** Flow cytometry was used to determine the changes in the cell cycle in *Lhpp* knockdown mESCs cultured with LIF-free medium or medium containing LIF for 3 days. **(F)** Quantification. All experiments were independently repeated at least three times, and the data are presented as the mean ± SD. **P* < 0.05, ***P* < 0.01, ****P* < 0.001. LHPP, phospholysine phosphohistidine inorganic pyrophosphate phosphatase; mESCs, mouse embryonic stem cells; DOX, doxycycline; LIF, leukemia inhibitory factor.

Supplementary Figure 5 Effects of *Lhpp* overexpression or knockdown on gene expressions in mESCs. **(A,B)** The mRNA levels of h*Lhpp*, m*Lhpp*, β*-catenin*, *CDK4*, *CyclinD1*, *P21* and *P27* **(A)** in mESCs treated with DOX (1 μg/ml) for 72 h and **(B)** in *Lhpp*-silenced mESCs cultured for 72 h, respectively, were detected by qPCR. **P* < 0.05, ***P* < 0.01. LHPP, phospholysine phosphohistidine inorganic pyrophosphate phosphatase; h*Lhpp*, human *Lhpp*; m*Lhpp*, mouse *Lhpp*; mESCs, mouse embryonic stem cells; DOX, doxycyclin.

## References

[B1] Abu-Dawud R. Graffmann N. Ferber S. Wruck W. Adjaye J. (2018). Pluripotent stem cells: induction and self-renewal. *Philos. Trans. R. Soc. Lond. B Biol. Sci*. 373:20170213. 10.1098/rstb.2017.0213 29786549 PMC5974437

[B2] Adamo A. Sese B. Boue S. Castano J. Paramonov I. Barrero M. J. (2011). LSD1 regulates the balance between self-renewal and differentiation in human embryonic stem cells. *Nat. Cell Biol*. 13 652–659. 10.1038/ncb2246 21602794

[B3] Boyer P. D. Deluca M. Ebner K. E. Hultquist D. E. Peter J. B. (1962). Identification of phosphohistidine in digests from a probable intermediate of oxidative phosphorylation. *J. Biol. Chem*. 237 PC3306–PC3308. 14014715

[B4] Cassar P. A. Stanford W. L. (2012). Integrating post-transcriptional regulation into the embryonic stem cell gene regulatory network. *J. Cell. Physiol*. 227 439–449. 10.1002/jcp.22787 21503874

[B5] Clevers H. Nusse R. (2012). Wnt/beta-catenin signaling and disease. *Cell* 149 1192–1205. 10.1016/j.cell.2012.05.012 22682243

[B6] Converge consortium. (2015). Sparse whole-genome sequencing identifies two loci for major depressive disorder. *Nature* 523 588–591. 10.1038/nature14659 26176920 PMC4522619

[B7] Dogan A. (2018). Embryonic stem cells in development and regenerative medicine. *Adv. Exp. Med. Biol*. 1079 1–15. 10.1007/5584_2018_175 29464659

[B8] Fuhs S. R. Meisenhelder J. Aslanian A. Ma L. Zagorska A. Stankova M. (2015). Monoclonal 1- and 3-phosphohistidine antibodies: new tools to study histidine phosphorylation. *Cell* 162 198–210. 10.1016/j.cell.2015.05.046 26140597 PMC4491144

[B9] Gabut M. Samavarchi-Tehrani P. Wang X. Slobodeniuc V. O’Hanlon D. Sung H. K. (2011). An alternative splicing switch regulates embryonic stem cell pluripotency and reprogramming. *Cell* 147 132–146. 10.1016/j.cell.2011.08.023 21924763

[B10] Gutierrez-Camino A. Martin-Guerrero I. Garcia-Orad A. (2017). Genetic susceptibility in childhood acute lymphoblastic leukemia. *Med. Oncol*. 34:179. 10.1007/s12032-017-1038-7 28905228

[B11] Han H. Irimia M. Ross P. J. Sung H. K. Alipanahi B. David L. (2013). MBNL proteins repress ES-cell-specific alternative splicing and reprogramming. *Nature* 498 241–245. 10.1038/nature12270 23739326 PMC3933998

[B12] Hindupur S. K. Colombi M. Fuhs S. R. Matter M. S. Guri Y. Adam K. (2018). The protein histidine phosphatase LHPP is a tumour suppressor. *Nature* 555 678–682. 10.1038/nature26140 29562234 PMC6376988

[B13] Huang G. Ye S. Zhou X. Liu D. Ying Q. L. (2015). Molecular basis of embryonic stem cell self-renewal: from signaling pathways to pluripotency network. *Cell. Mol. Life Sci*. 72 1741–1757. 10.1007/s00018-015-1833-2 25595304 PMC4809369

[B14] Iacovino M. Roth M. E. Kyba M. (2014). Rapid genetic modification of mouse embryonic stem cells by inducible cassette exchange recombination. *Methods Mol. Biol*. 1101 339–351. 10.1007/978-1-62703-721-1_16 24233789 PMC3935508

[B15] Kyba M. Perlingeiro R. C. Daley G. Q. (2002). HoxB4 confers definitive lymphoid-myeloid engraftment potential on embryonic stem cell and yolk sac hematopoietic progenitors. *Cell* 109 29–37. 10.1016/s0092-8674(02)00680-3 11955444

[B16] Li L. Zhang L. Liu G. Feng R. Jiang Y. Yang L. (2014). Synergistic transcriptional and post-transcriptional regulation of ESC characteristics by core pluripotency transcription factors in protein-protein interaction networks. *PLoS One* 9:e105180. 10.1371/journal.pone.0105180 25171496 PMC4149371

[B17] Liou J. Y. Ko B. S. Chang T. C. (2017). An efficient transfection method for differentiation and cell proliferation of mouse embryonic stem cells. *Methods Mol. Biol*. 1622 139–147. 10.1007/978-1-4939-7108-4_11 28674807

[B18] Liu X. Yao Y. Ding H. Han C. Chen Y. Zhang Y. (2016). USP21 deubiquitylates Nanog to regulate protein stability and stem cell pluripotency. *Signal Transduct. Target. Ther*. 1:16024. 10.1038/sigtrans.2016.24 29263902 PMC5661642

[B19] Livak K. J. Schmittgen T. D. (2001). Analysis of relative gene expression data using real-time quantitative PCR and the 2(-Delta Delta C(T)) method. *Methods* 25 402–408. 10.1006/meth.2001.1262 11846609

[B20] Polimanti R. Wang Q. Meda S. A. Patel K. T. Pearlson G. D. Zhao H. (2017). The interplay between risky sexual behaviors and alcohol dependence: genome-wide association and neuroimaging support for LHPP as a risk gene. *Neuropsychopharmacology* 42 598–605. 10.1038/npp.2016.153 27531626 PMC5240175

[B21] Ran X. Xiao C. H. Xiang G. M. Ran X. Z. (2017). Regulation of embryonic stem cell self-renewal and differentiation by microRNAs. *Cell. Reprogram*. 19 150–158. 10.1089/cell.2016.0048 28277752

[B22] Sampath P. Pritchard D. K. Pabon L. Reinecke H. Schwartz S. M. Morris D. R. (2008). A hierarchical network controls protein translation during murine embryonic stem cell self-renewal and differentiation. *Cell Stem Cell* 2 448–460. 10.1016/j.stem.2008.03.013 18462695

[B23] Selvaraj P. Xiao L. Lee C. Murthy S. R. Cawley N. X. Lane M. (2017). Neurotrophic factor-alpha1: a key Wnt-beta-catenin dependent anti-proliferation factor and ERK-Sox9 activated inducer of embryonic neural stem cell differentiation to astrocytes in neurodevelopment. *Stem Cells* 35 557–571. 10.1002/stem.2511 27709799 PMC6131007

[B24] Si Y. Zhu J. Huang X. Zhu P. Xie C. (2016). Effects of *Panax notoginseng* saponins on proliferation and differentiation of rat embryonic cortical neural stem cells. *J. Chin. Med. Assoc*. 79 256–263. 10.1016/j.jcma.2015.10.011 26915440

[B25] Srivastava S. Li Z. Soomro I. Sun Y. Wang J. Bao L. (2018). Regulation of KATP channel trafficking in pancreatic beta-cells by protein histidine phosphorylation. *Diabetes* 67 849–860. 10.2337/db17-1433 29440278 PMC5909995

[B26] Tao J. Zhang R. Singh S. Poddar M. Xu E. Oertel M. (2017). Targeting beta-catenin in hepatocellular cancers induced by coexpression of mutant beta-catenin and K-Ras in mice. *Hepatology* 65 1581–1599. 10.1002/hep.28975 27981621 PMC5397318

[B27] Taracha A. Kotarba G. Wilanowski T. (2017). [Methods of analysis of protein phosphorylation]. *Postepy Biochem*. 63 137–142. 28689381

[B28] Vijayakrishnan J. Kumar R. Henrion M. Y. Moorman A. V. Rachakonda P. S. Hosen I. (2017). A genome-wide association study identifies risk loci for childhood acute lymphoblastic leukemia at 10q26.13 and 12q23.1. *Leukemia* 31 573–579. 10.1038/leu.2016.271 27694927 PMC5336191

[B29] Wang S. Li Z. Shen H. Zhang Z. Yin Y. Wang Q. (2016). Quantitative phosphoproteomic study reveals that protein kinase a regulates neural stem cell differentiation through phosphorylation of catenin Beta-1 and glycogen synthase kinase 3beta. *Stem Cells* 34 2090–2101. 10.1002/stem.2387 27097102

[B30] Watanabe N. Osada H. (2016). Small molecules that target phosphorylation dependent protein-protein interaction. *Bioorg. Med. Chem*. 24 3246–3254. 10.1016/j.bmc.2016.03.023 27017542

[B31] Wen J. Lv R. Ma H. Shen H. He C. Wang J. (2018). Zc3h13 regulates nuclear RNA m(6)A methylation and mouse embryonic stem cell self-renewal. *Mol. Cell* 69 1028–1038.e6. 10.1016/j.molcel.2018.02.015 29547716 PMC5858226

[B32] Whyte W. A. Bilodeau S. Orlando D. A. Hoke H. A. Frampton G. M. Foster C. T. (2012). Enhancer decommissioning by LSD1 during embryonic stem cell differentiation. *Nature* 482 221–225. 10.1038/nature10805 22297846 PMC4144424

[B33] Xu Z. Robitaille A. M. Berndt J. D. Davidson K. C. Fischer K. A. Mathieu J. (2016). Wnt/beta-catenin signaling promotes self-renewal and inhibits the primed state transition in naive human embryonic stem cells. *Proc. Natl. Acad. Sci. U.S.A*. 113 E6382–E6390. 10.1073/pnas.1613849113 27698112 PMC5081574

[B34] Yang J. Mowry L. E. Nejak-Bowen K. N. Okabe H. Diegel C. R. Lang R. A. (2014). beta-catenin signaling in murine liver zonation and regeneration: a Wnt-Wnt situation! *Hepatology* 60 964–976. 10.1002/hep.27082 24700412 PMC4139486

[B35] Yokoi F. Hiraishi H. Izuhara K. (2003). Molecular cloning of a cDNA for the human phospholysine phosphohistidine inorganic pyrophosphate phosphatase. *J. Biochem*. 133 607–614. 10.1093/jb/mvg078 12801912

[B36] Zhang T. Lin Y. Liu J. Zhang Z. G. Fu W. Guo L. Y. (2016). Rbm24 regulates alternative splicing switch in embryonic stem cell cardiac lineage differentiation. *Stem Cells* 34 1776–1789. 10.1002/stem.2366 26990106

[B37] Zhao H. Han Z. Liu X. Gu J. Tang F. Wei G. (2017). The chromatin remodeler Chd4 maintains embryonic stem cell identity by controlling pluripotency- and differentiation-associated genes. *J. Biol. Chem*. 292 8507–8519. 10.1074/jbc.M116.770248 28298436 PMC5437254

[B38] Zheng J. Dai X. Chen H. Fang C. Chen J. Sun L. (2018). Down-regulation of LHPP in cervical cancer influences cell proliferation, metastasis and apoptosis by modulating AKT. *Biochem. Biophys. Res. Commun*. 503 1108–1114. 10.1016/j.bbrc.2018.06.127 29944886

