# Supplementary figures and images for: LHPP-Mediated Histidine Dephosphorylation Suppresses the Self-Renewal of Mouse Embryonic Stem Cells
Source: Front Cell Dev Biol. 2021 Mar 16;9:638815. doi: 10.3389/fcell.2021.638815 (PMC8007871; doi:10.3389/fcell.2021.638815)

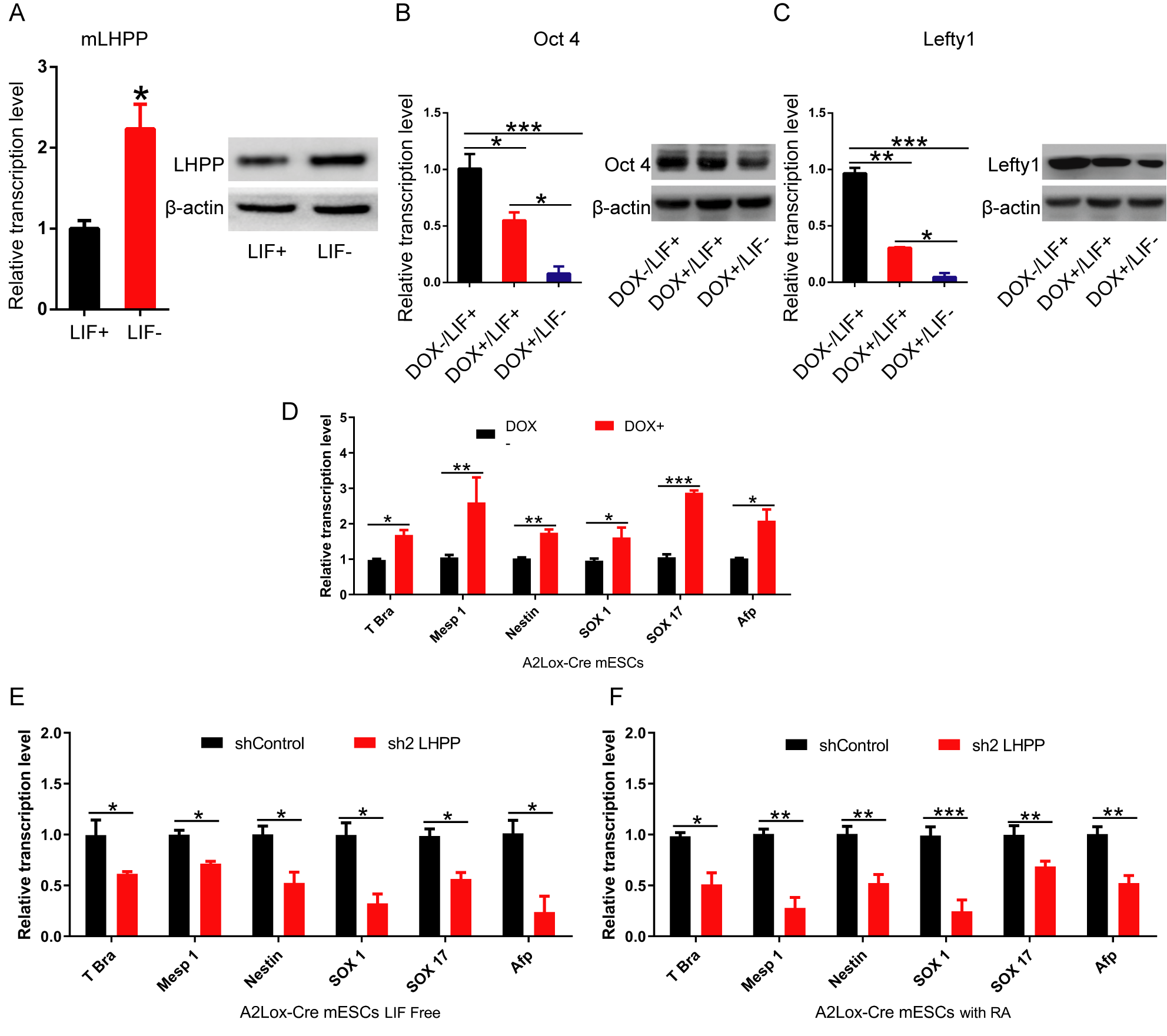

Supplement: Supplementary Figure 1 — The effects of LHPP overexpression or silencing on gene expressions. (A–C) qPCR and western blotting quantification of (A) mLhpp, (B) Oct4, and (C) Lefty1 levels in A2Lox-Cre mESCs treated with LIF alone or in combination with DOX (1 μg/ml). (D) qPCR quantification of the mRNA levels of SOX17, Afp, T-Bra, Mesp1, Nestin, and SOX1 in mESCs treated with LIF alone or in combination with DOX (1 μg/ml). (E,F) qPCR detection of the mRNA levels of SOX17, Afp, T-Bra, Mesp1, Nestin and SOX1 (E) in mESCs transfected with shRNA targeting Lhpp, or (F) in A2Lox-Cre mESCs treated with RA (10 mM). All experiments were independently repeated at least three times, and the data are represented as the mean ± SD. ∗P < 0.05, **P < 0.01, ***P < 0.001. LHPP, phospholysine phosphohistidine inorganic pyrophosphate phosphatase; mLHPP, mouse LHPP; mESCs, mouse embryonic stem cells; DOX, doxycycline; LIF, leukemia inhibitory factor; RA, retinoic acid. [file Image_1.TIF]

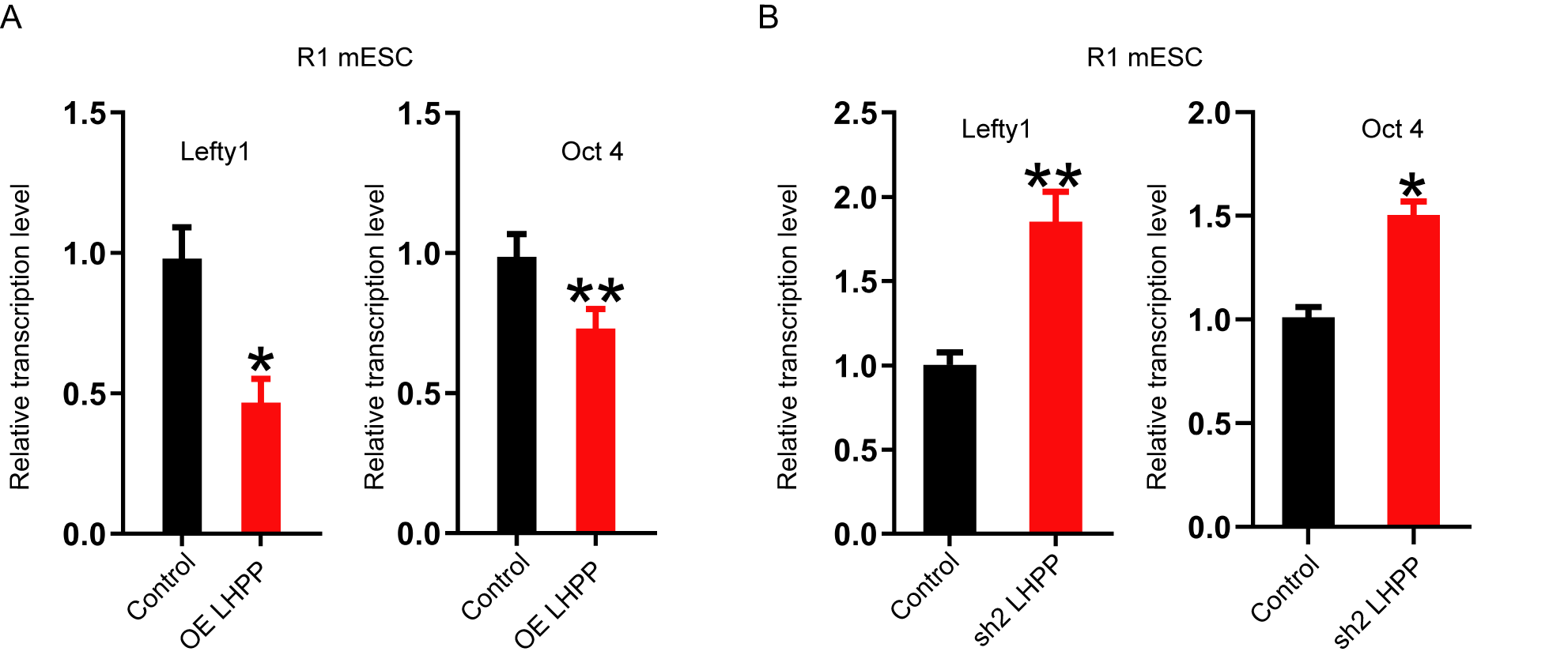

Supplement: Supplementary Figure 2 — The expression of Lefty1 and Oct4 in Lhpp-overexpressed and silenced R1 mESCs. (A) Lefty1 and Oct4 mRNA expression were downregulated in Lhpp-overexpressed R1 mESCs. (B) Lefty1 and Oct4 mRNA expression were upregulated in L Lhpp-silenced R1 mESCs. All experiments were independently repeated at least three times, and the data are represented as the mean ± SD. *P < 0.05, **P < 0.01. LHPP, phospholysine phosphohistidine inorganic pyrophosphate phosphatase; mESCs, mouse embryonic stem cells. [file Image_2.TIF]

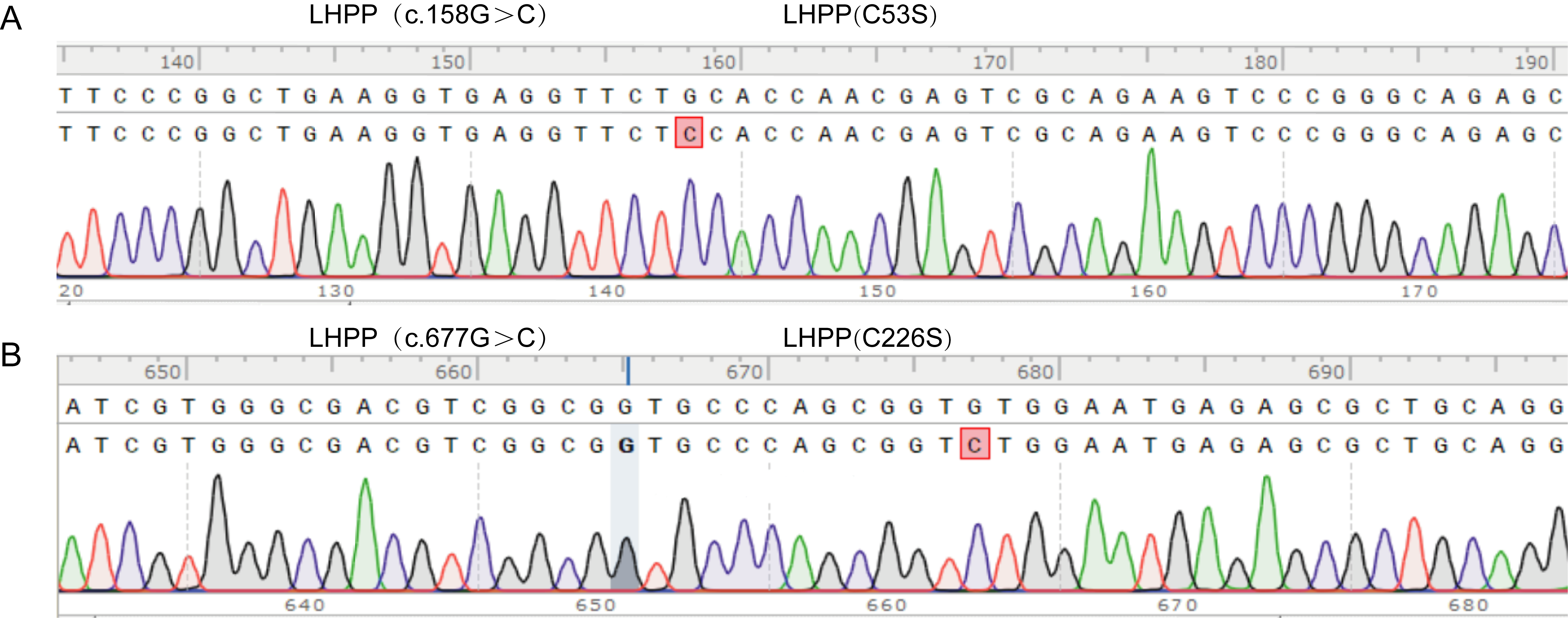

Supplement: Supplementary Figure 3 — Schematic diagram of the mutation sites in Lhpp. (A) Sanger sequencing confirmed the successful mutation of LHPP at position 158, labeled as LHPP (c.158G > C). The corresponding amino acid sequence is LHPP (C53S). (B) Sanger sequencing showed the successful mutation of LHPP at position 677, labeled LHPP (c.677G > C). The corresponding amino acid sequence is LHPP (C226S). The mutation sites are colored in red. [file Image_3.TIF]

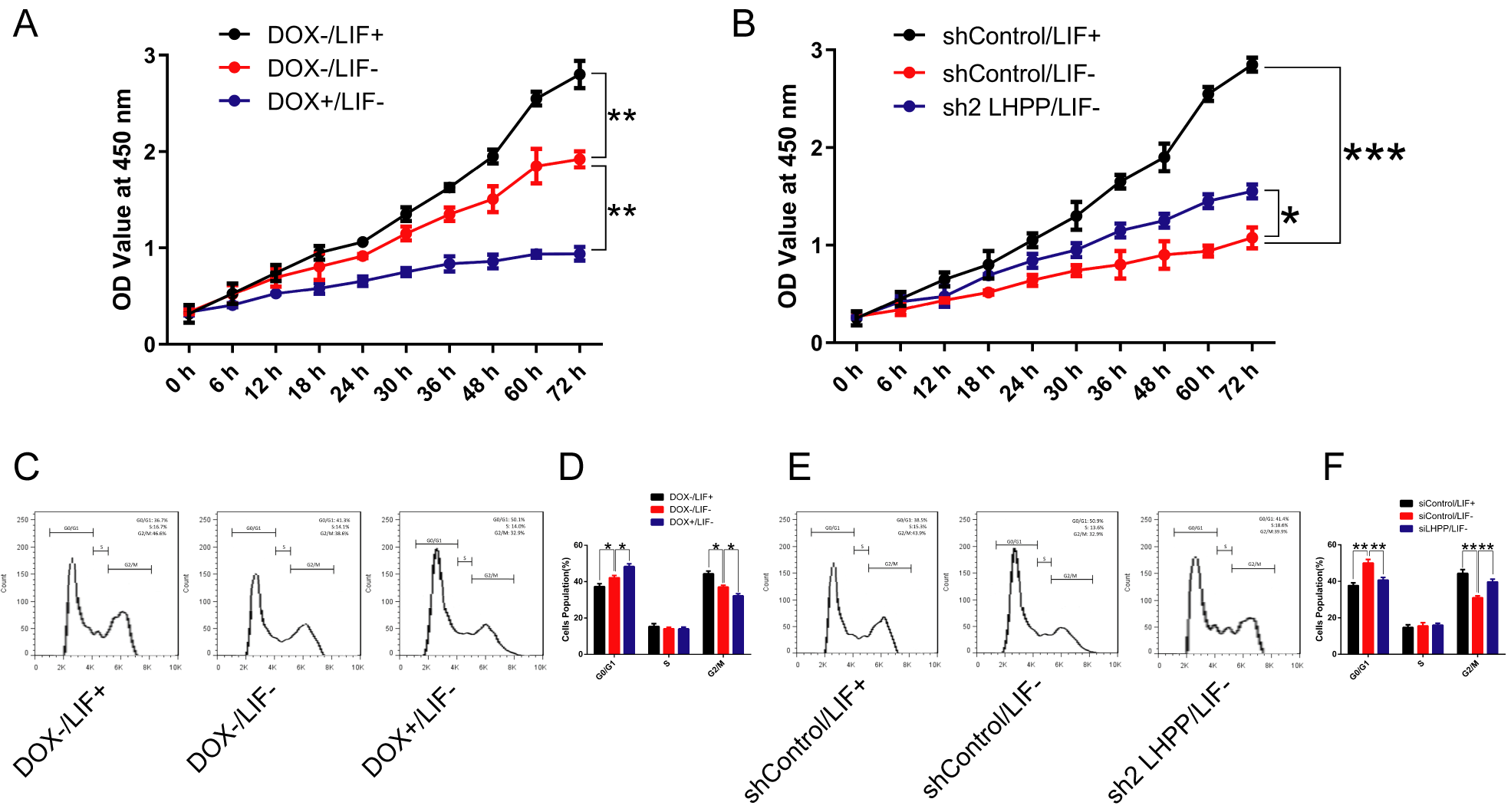

Supplement: Supplementary Figure 4 — Effect of LHPP on proliferation and cell cycle of mESCs. (A,B) Cell Counting Kit-8 assay was used to detect the proliferation of (A) A2Lox-Cre mESCs following treatment with DOX (1 μg/ml) alone or in combination with LIF and (B) Lhpp knockdown mESCs cultured with LIF-free medium. (C,D) Flow cytometry was used to detect changes to the cell cycle in mESCs cultured with LIF-free medium, with or without DOX (1 μg/ml) for 3 days, respectively. (D) Quantification. (E,F) Flow cytometry was used to determine the changes in the cell cycle in Lhpp knockdown mESCs cultured with LIF-free medium or medium containing LIF for 3 days. (F) Quantification. All experiments were independently repeated at least three times, and the data are presented as the mean ± SD. *P < 0.05, **P < 0.01, ***P < 0.001. LHPP, phospholysine phosphohistidine inorganic pyrophosphate phosphatase; mESCs, mouse embryonic stem cells; DOX, doxycycline; LIF, leukemia inhibitory factor. [file Image_4.TIF]

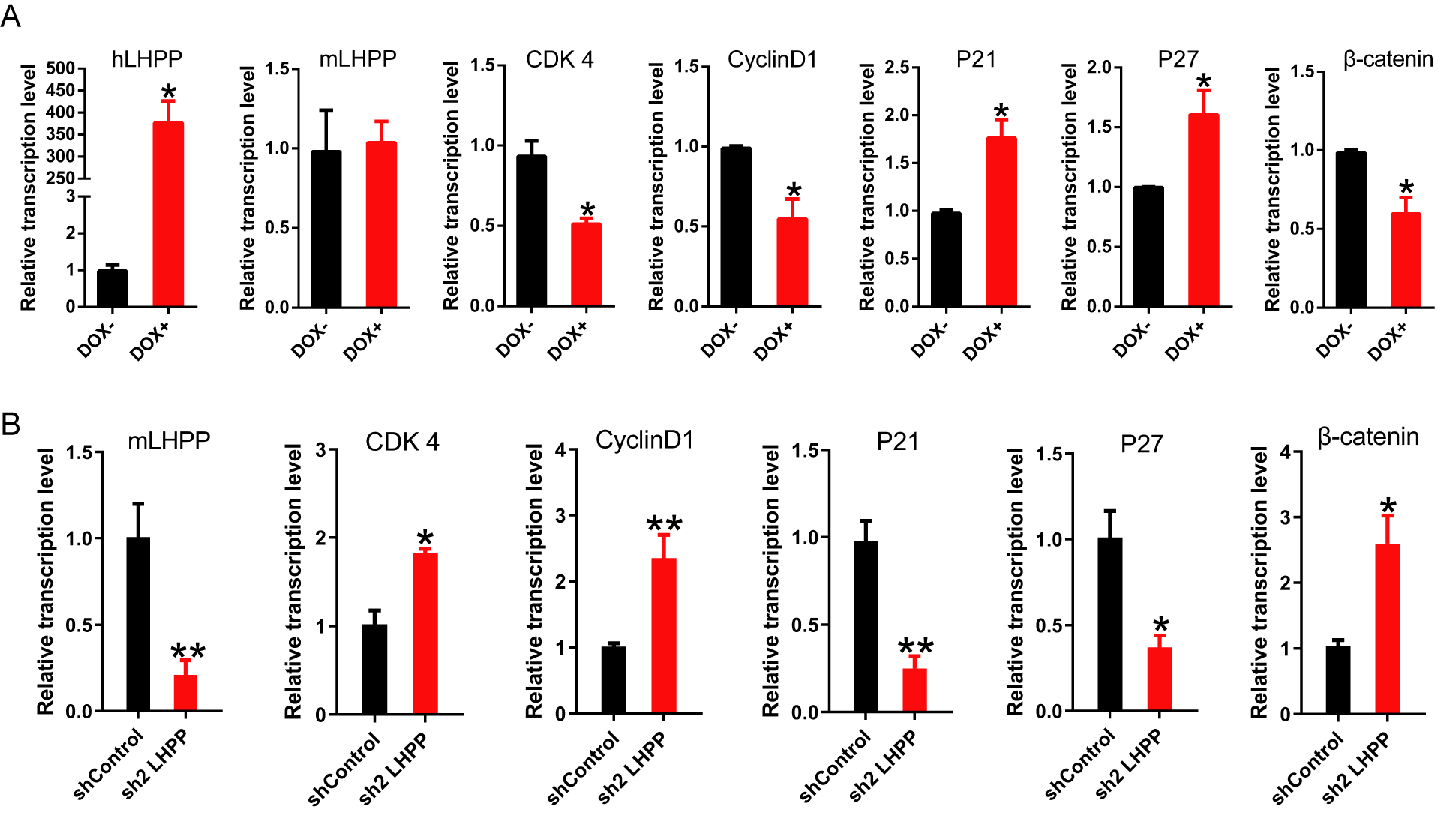

Supplement: Supplementary Figure 5 — Effects of Lhpp overexpression or knockdown on gene expressions in mESCs. (A,B) The mRNA levels of hLhpp, mLhpp, β-catenin, CDK4, CyclinD1, P21 and P27 (A) in mESCs treated with DOX (1 μg/ml) for 72 h and (B) in Lhpp-silenced mESCs cultured for 72 h, respectively, were detected by qPCR. *P < 0.05, **P < 0.01. LHPP, phospholysine phosphohistidine inorganic pyrophosphate phosphatase; hLhpp, human Lhpp; mLhpp, mouse Lhpp; mESCs, mouse embryonic stem cells; DOX, doxycyclin. [file Image_5.TIF]

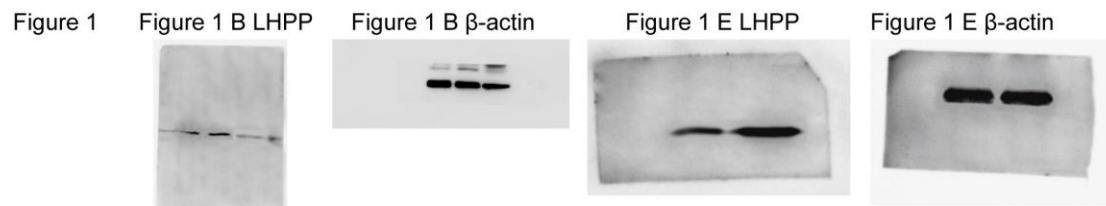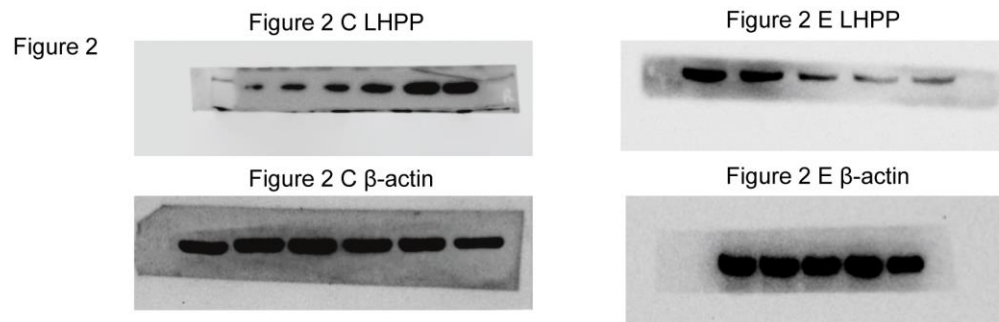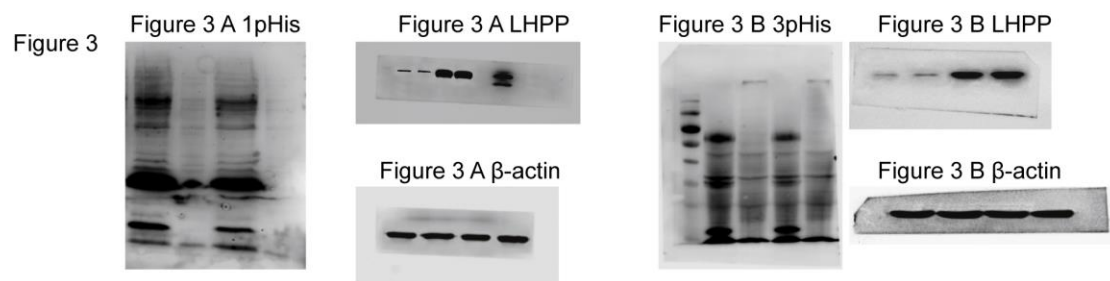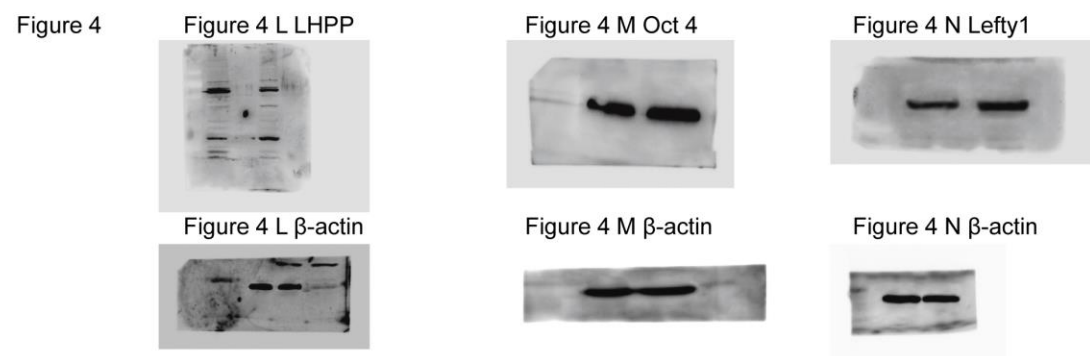

Figure 5

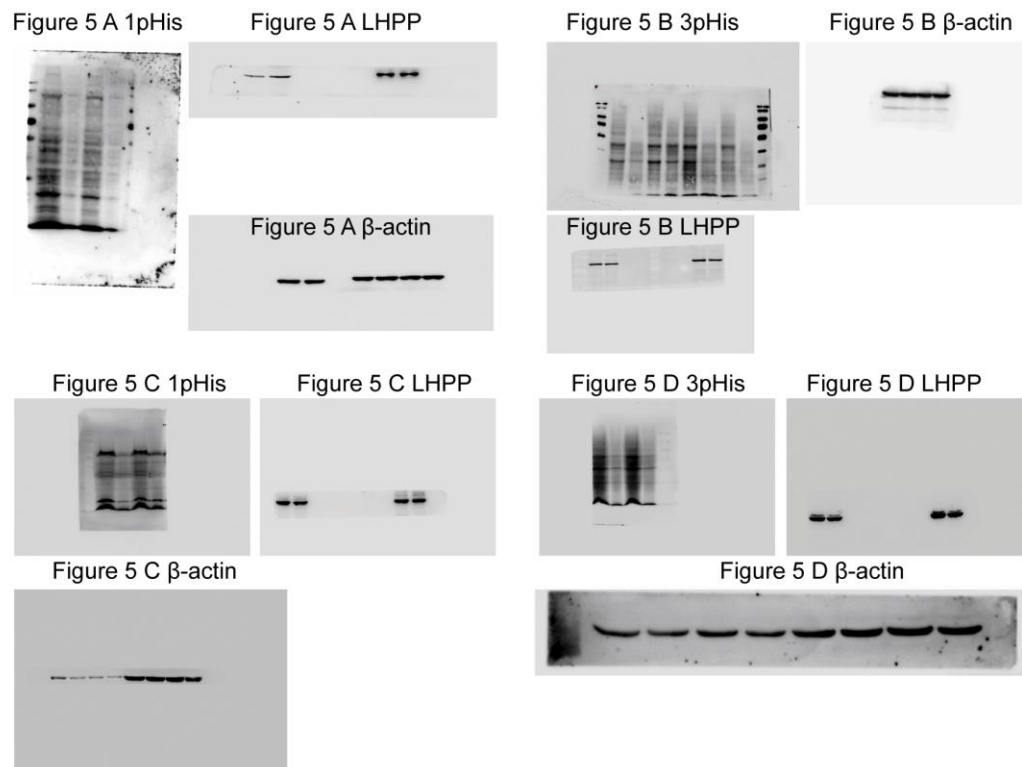

Figure 7

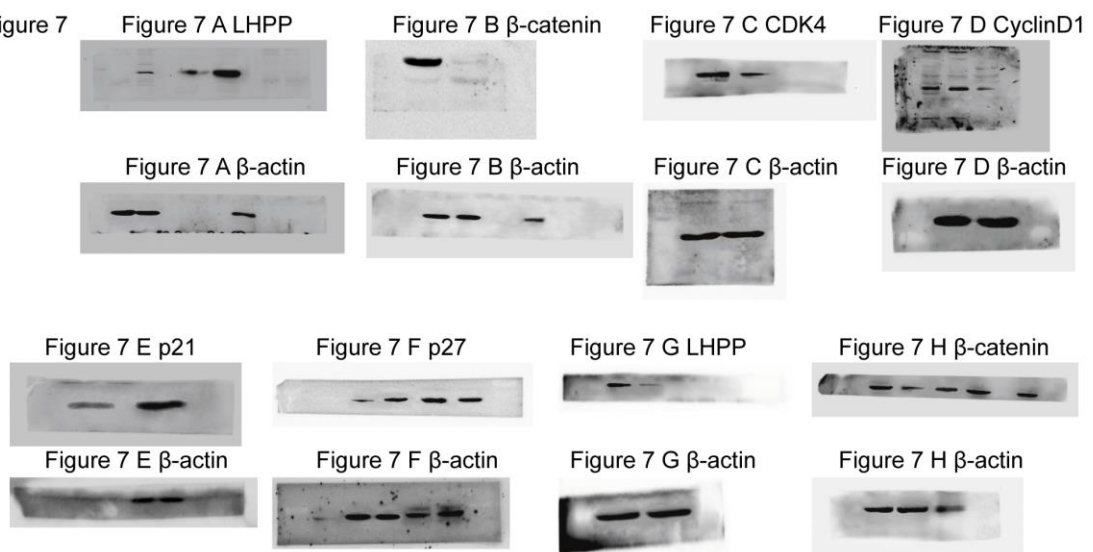

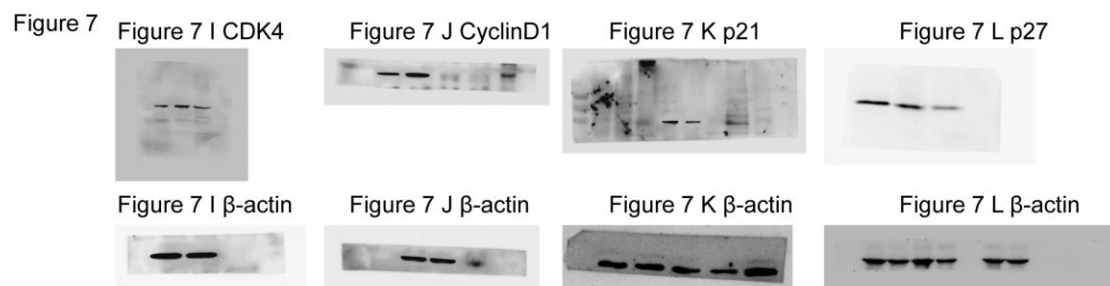

Supplementary Figure 1

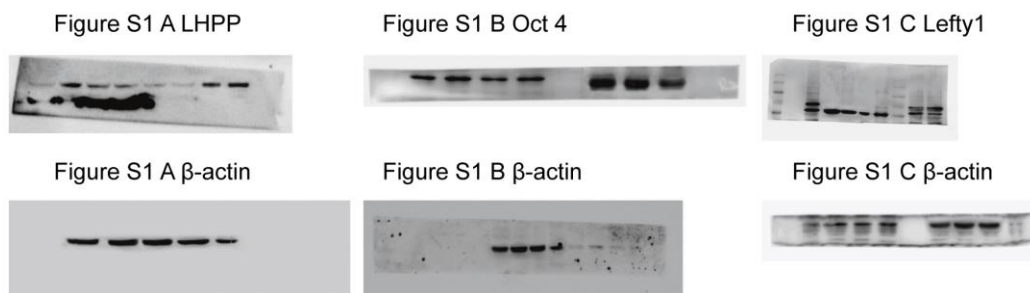

Supplement: Supplementary file 6 [file Data_Sheet_1.PDF]
